# [^18^F]F-DCFPyL PET/MRI radiomics for intraprostatic prostate cancer detection and metastases prediction using whole-gland segmentation

**DOI:** 10.1093/bjr/tqaf014

**Published:** 2025-01-23

**Authors:** Seyed Ali Mirshahvalad, Adriano Basso Dias, Claudia Ortega, Jorge Andres Abreu Gomez, Satheesh Krishna, Nathan Perlis, Alejandro Berlin, Theodorus van der Kwast, Kartik Jhaveri, Sangeet Ghai, Ur Metser, Anna Theresa Santiago, Patrick Veit-Haibach

**Affiliations:** Joint Department of Medical Imaging, University Medical Imaging Toronto (UMIT), University Health Network, Mount Sinai Hospital & Women’s College Hospital; University of Toronto, Toronto, ON, M5G 2M9, Canada; Joint Department of Medical Imaging, University Medical Imaging Toronto (UMIT), University Health Network, Mount Sinai Hospital & Women’s College Hospital; University of Toronto, Toronto, ON, M5G 2M9, Canada; Joint Department of Medical Imaging, University Medical Imaging Toronto (UMIT), University Health Network, Mount Sinai Hospital & Women’s College Hospital; University of Toronto, Toronto, ON, M5G 2M9, Canada; Joint Department of Medical Imaging, University Medical Imaging Toronto (UMIT), University Health Network, Mount Sinai Hospital & Women’s College Hospital; University of Toronto, Toronto, ON, M5G 2M9, Canada; Joint Department of Medical Imaging, University Medical Imaging Toronto (UMIT), University Health Network, Mount Sinai Hospital & Women’s College Hospital; University of Toronto, Toronto, ON, M5G 2M9, Canada; Division of Urology, Department of Surgery, Princess Margaret Cancer Centre, University Health Network, Toronto, ON, M5G 2M9, Canada; Department of Radiation Oncology, Princess Margaret Cancer Center, University of Toronto, Toronto, ON, M5G 2M9, Canada; Laboratory Medicine Program, University Health Network, Toronto, ON, M5G 2M9, Canada; Joint Department of Medical Imaging, University Medical Imaging Toronto (UMIT), University Health Network, Mount Sinai Hospital & Women’s College Hospital; University of Toronto, Toronto, ON, M5G 2M9, Canada; Joint Department of Medical Imaging, University Medical Imaging Toronto (UMIT), University Health Network, Mount Sinai Hospital & Women’s College Hospital; University of Toronto, Toronto, ON, M5G 2M9, Canada; Joint Department of Medical Imaging, University Medical Imaging Toronto (UMIT), University Health Network, Mount Sinai Hospital & Women’s College Hospital; University of Toronto, Toronto, ON, M5G 2M9, Canada; Biostatistics Department, Princess Margaret Cancer Centre, University Health Network, Toronto, ON, M5G 2M9, Canada; Joint Department of Medical Imaging, University Medical Imaging Toronto (UMIT), University Health Network, Mount Sinai Hospital & Women’s College Hospital; University of Toronto, Toronto, ON, M5G 2M9, Canada

**Keywords:** prostate, PSMA, DCFPyL, positron emission tomography, MRI, radiomics

## Abstract

**Objectives:**

To evaluate [^18^F]F-DCFPyL PET/MRI whole-gland-derived radiomics for detecting clinically significant (cs) prostate cancer (PCa) within the prostate gland and predicting extra-prostatic metastasis (N and M staging).

**Methods:**

In this single-centre, retrospective study, therapy-naïve PCa patients who underwent [^18^F]F-DCFPyL PET/MRI were included. Whole-prostate segmentation was performed. Feature extraction from each modality was done. The selection of potential variables was made through regularized binomial logistic regression. The oversampled training data were used to train binomial logistic regression for each outcome. The estimates of the models were calculated, and the mean accuracy was reported. The trained models were assessed on the test data for comparative evaluation of performance.

**Results:**

A total of 103 patients (mean age = 65; mean PSA = 23.4) were studied. Among them, 89 had csPCa and 20 had metastatic disease. There were five radiomics variables selected for the International Society of Urological Pathology Grade Group (ISUP GG) ≥ 2 from T2w, ADC, and PET. To detect N1, five radiomics variables were selected from the T2w and PET. For M1, four radiomics variables were selected from T2w and ADC. Regarding the performance of models for the prediction of csPCa, the imaging-based hybrid model (T2w + PET) provided the highest AUC (0.98). The performance of N1 models showed the highest AUC (0.80) for T2w + PET. To predict M1, the T2w + ADC model showed the highest AUC (0.93).

**Conclusions:**

Whole-gland PET/MRI radiomics may provide a reliable model to predict csPCa. Also, acceptable performance was reached for predicting metastatic disease in our limited population. Our findings may support the value of whole-gland radiomics for non-invasive csPCa detection and prediction of metastatic disease.

**Advances in knowledge:**

Whole-gland PET/MRI radiomics, a less operator-dependent segmentation method, can be potentially used for treatment personalization in PCa patients.

**Trial Registration:**

NCT03535831. Registered 2018; NCT03149861. Registered 2017

## Introduction

Prostate cancer (PCa) is the most common malignancy and the second most common cause of cancer-related mortality in the male population.^1^ The diagnosis is usually made by histopathologic evaluation after a targeted or systematic biopsy, most commonly performed after detecting an elevated serum prostate-specific antigen (PSA) level.^2^ Currently, the 5-scale International Society of Urological Pathology Grade Group (ISUP GG) is used to classify patients into different risk groups based on their Gleason score (GS).^3^ This pathological group classification has clinical importance (eg, men with ISUP GG1 mostly undergo active surveillance, while men with ISUP ≥ 2 often benefit from radical treatment).

MRI is considered the imaging technique of choice for detecting clinically significant (cs) PCa (ISUP GG ≥ 2), even in biopsy-naïve patients, compared to systematic TRUS biopsies.^4^ Nevertheless, MRI also may miss approximately 10% of clinically significant csPCa, though there are some mixed results about how much is really missed, and some studies reported higher proportions.^5^^,^^6^ Thus, further improvement of non-invasive imaging techniques is needed to improve the detection of csPCa.^7^

More recently, positron emission tomography (PET) using prostate-specific membrane antigen (PSMA), an overexpressed transmembrane cell-surface protein in the PCa cells, has been extensively evaluated in different scenarios of the PCa workup.^8–10^ PSMA can be targeted by different PET tracers to visualize PCa lesions. [^18^F]F-DCFPyL (2-(3-(1-carboxy-5-[(6-^18^F-fluoro-pyridine-3-carbonyl)-amino]-pentyl)-ureido)-pentanedioic acid), a second-generation FDA-approved PSMA tracer, has demonstrated accurate results in various clinical scenarios.^11–14^ By merging the advantages of PSMA PET and MRI as a hybrid modality, PSMA PET/MRI can be used in the evaluation of patients with PCa.^14–17^

In combination with clinical data, radiomics is a non-invasive method that has shown promising results in medical imaging, particularly in oncology.^18^^,^^19^ PSMA PET/MRI-derived radiomic features have shown to be capable of improving PSMA PET/MRI baseline diagnostic accuracy.^20–22^ In this study, we aimed to evaluate the value of [^18^F]F-DCFPyL PET/MRI-derived radiomic features in detecting intraprostatic csPCa and predicting nodal and distant metastases using a whole-gland approach.

## Methods

### Patient data

This ethics review board-approved, retrospective analysis of two prospective clinical trials (ClinicalTrials.gov Identifiers: NCT03535831 [Cohort A] and NCT03149861 [Cohort B]) included 103 treatment-naïve men who had confirmed or suspected PCa.^23^ It received approval from the joint department of the medical imaging department, followed institutional guidelines and regulations, and was done in accordance with the Declaration of Helsinki. Three patient groups were recruited: patients with unfavourable intermediate or high-risk PCa (*n* = 52), patients being considered for focal therapy (*n* = 31), or those who had either suspicion of PCa and negative systematic biopsies or clinically discordant low-risk PCa (*n* = 20). All patients were enrolled between June 2017 and December 2020 and underwent [^18^F]F-DCFPyL PET/MRI. Informed consent was obtained from all participants. Exclusion criteria included patients who received prior primary therapy, PCa with significant sarcomatoid or spindle cell or neuroendocrine differentiation, or contraindication to MRI or gadolinium as per departmental safety guidelines.

### Imaging protocol

[^18^F]F-DCFPyL was synthesized as previously described in the literature.^24^ PET images were acquired 118 (±20) min after intravenous administration of 324 (±12) MBq of [^18^F]F-DCFPyL on the PET/MRI platform. All scans were performed using Biograph mMR (Siemens Healthcare, Germany). The imaging protocol for PET/MRI was performed as previously documented.^25^ In summary, whole-body MRI (top of the skull to upper thighs) was imaged with axial Dixon sequences for attenuation correction and axial VIBE T1 post-contrast. Multiparametric MRI of the prostate was done as follows: multiplanar T2, axial DWI (*B*-values: 0, 50, 900, 1600 s/mm^2^), and T1-weighted volumetric interpolated breath-hold (VIBE) examination (15 phases, maximal temporal resolution <7 s for 1.26 min). The detailed MRI parameters are indicated in Tables S1 and S2. Whole-body PET was acquired with the same field of view as whole-body MRI (5-6 bed positions; 2-3 min/bed for whole-body acquisition). An additional dedicated bed was also acquired from the pelvis.

### Image segmentation and radiomic features extraction

Radiomic feature analysis of PET/MRI was obtained using LIFEx version 6.1 software (lifexsoft.org)^26^ via the quantitation of various radiomics features based on the spatial arrangement and variation of pixel intensities within a defined volume of interest. Whole-prostate segmentations were performed on each modality separately.^27^ On MRI, whole-gland T2-weighted sequence and whole-gland apparent diffusion coefficient (ADC) map were evaluated. Since a thresholding method was not available for the MRI component (T2, ADC), manual contouring was done for the MRI-derived volumes of interest (VOIs) (by a radiologist with five years of experience in prostate MRI) in a slice-by-slice fashion to cover the entire prostate (whole-gland segmentation). These VOIs were co-registered to the hybrid PET to contain the whole gland, and rechecked via background thresholding.

**Figure 1.**
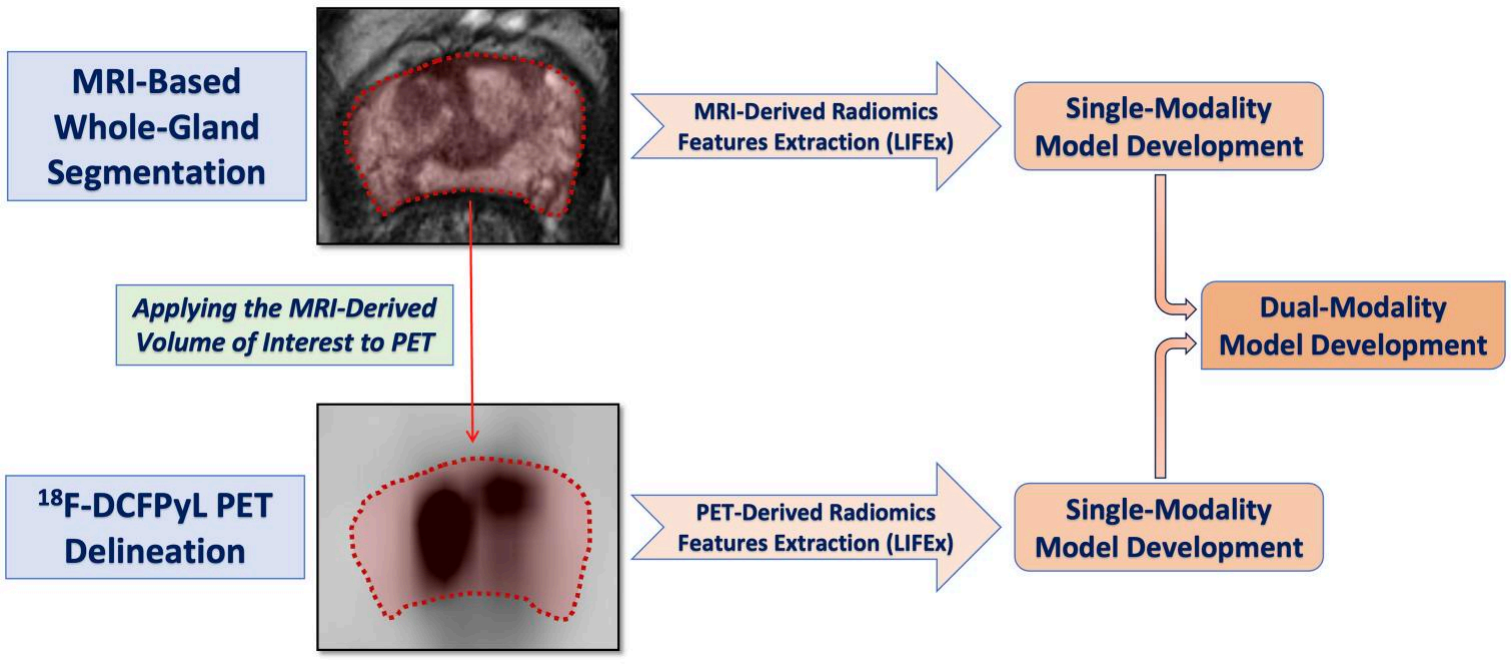
Segmentation process, feature extraction, and model creation in the study.

The radiomics features, including overall 307 different features (Table S3), were extracted from the segmented volumes in accordance with the image biomarker standardization initiative (IBSI) guidelines^28^ and included the following: conventional metrics, size and shape features, and textural features. The feature extraction workflow is illustrated in Figure 1. This methodology was used in previous publications.^29^^,^^30^

### Reference standards

Suspicious prostatic lesions were correlated with histopathology (prostate biopsy) findings in all patients. The highest reported ISUP GG was considered as the representative of each prostate gland. csPCa was defined as ISUP GG ≥ 2. For nodal and distant metastatic evaluation, a composite reference standard was applied to characterize all lesions identified on conventional imaging (CT and bone scan ± mpMRI) or PET and included histopathology, correlative imaging, and clinical/biochemical follow-up (Table S4). The prescription of ancillary imaging studies was at the discretion of the treating oncologist. For discrepant lesions, the definition of a true positive was based on the composite reference standard as assessed by two reviewers, with diagnostic criteria previously defined.^31–34^ Specifically, lymph nodes were considered positive if the typical distribution of PCa metastases (nodal spread in an ascending pathway from pelvic stations to common iliac nodes and the retroperitoneum), and/or size change on follow-up congruent with interval therapy. The typical appearance of multifocal metastatic bone lesions on any modality, or concordance on two different modalities for solitary or few lesions was considered confirmatory. Bone lesions in men with undetectable PSA after radical primary therapy only were considered benign. For suspected visceral metastases, in the absence of histological proof, correlative imaging or morphological change on follow-up imaging congruent with interval therapy was used for confirmation. Lymph nodes and skeletal and visceral lesions which did not fulfil the criteria for malignancy on both conventional imaging and PET were deemed true negative. The clinical stage for pelvic nodal (N) and distant metastases (M) following the American Joint Committee on Cancer [AJCC] Cancer Staging 8th edition were tabulated for conventional imaging and PET.^35^ Equivocal lesions on PET/MRI were considered negative.

### Statistical analysis

Patient clinical characteristics and outcomes were summarized as means (standard deviation) or numbers (percentages). Data were split into 70% training data (*n* = 73) and 30% test data (*n* = 30) based on outcome. T2, ADC, and PET radiomics variables in the training data were pre-processed through standardization (centred and scaled), exclusion of zero or near-zero variance variables, and identification and exclusion of correlated predictors based on a pair-wise Pearson correlation cut-off of 0.75 indicating strong correlation. A total of 56 and 55 radiomic variables were assessed as possible predictors for ISUP GG ≥ 2, N stage and M stage, respectively (Appendices XX-1 and XX-2). The test data were centered and scaled using the pre-processing parameters of the training data. The selection of potential radiomics variables in the training data for each outcome was done through regularized binomial logistic regression using the least absolute shrinkage and selection operator (LASSO) according to the minimum lambda from 10-fold cross-validation. Imbalance in the ISUP GG ≥ 2, N stage and M stage outcomes was addressed using synthetic minority oversampling technique (SMOTE) to oversample the minority classes in the training data to yield up to 1:1 balance with the majority class. The oversampled training data were used to train binomial logistic regression models for each outcome including a base clinical model for age and PSA, single imaging modality (T2, ADC, or PET) models, and age- and PSA-adjusted models through 10-fold cross-validation repeated five times. The estimates of the odds ratios and Akaike information criterion (AIC) of the final models trained and the mean accuracy were reported. The trained models were assessed on the test data for comparative evaluation of performance based on sensitivity, specificity, positive predictive value, negative predictive value, and the area under the curve (AUC) of the receiver operating characteristic. The classification threshold using the Youden index for single imaging modality models with one variable was reported. Pair-wise AUC comparisons were done using DeLong’s test. Statistical analysis was done using the tidyverse (version 2.0.0), corrplot (version 0.92), glmnet (version 4.1-8), caret (version 6.0-94), epiR (version 2.0.70), and pROC (1.18.5) libraries in R version 4.3.1 (R Core Team, 2023).

## Results

A total of 103 patients [mean age (SD) = 65.0 (8.1); mean PSA (SD) = 23.4 (42.3), Table 1] were studied. Based on the histopathologic evaluation, 10 patients had negative results for PCa, four had ISUP GG1, and 89 men had csPCa (ISUP GG ≥ 2). In total, 20 and eight patients had lymph node (N1) and distant metastasis (M1), respectively. Detailed reference standards’ results for N and M staging are provided in Table S4. Follow-up data were available for 51 men, with a median follow-up of 18 months (range: 1-31 months). There were five radiomics variables selected for ISUP GG ≥ 2 from the T2w (T2w GLZLM ZLNU), ADC (ADC CONVENTIONAL ExcessKurtosis, ADC GLCM Homogeneity InverseDifference), and PET (PET DISCRETIZED SUV, PET NGLDM Busyness) modalities. To predict N1 disease, there were five radiomics variables selected from the T2w (T2 CONVENTIONAL Skewness, T2 CONVENTIONAL Kurtosis, T2 GLRLM LRE) and PET (PET GLRLM LRHGE, PET70% DISCRETIZED TLG) modalities. For M staging prediction (M1), there were four radiomics variables selected from the T2w (T2 DISCRETIZED Q2, T2 DISCRETIZED Skewness, T2 DISCRETIZED HISTO ExcessKurtosis) and ADC (ADC GLRLM SRE) sequences.

**Table 1.** Patients’ characteristics (*n* = 103).

Characteristic	Value
Age, y, Mean (SD)	65.0 (8.1)
PSA level, ng/mL, Mean (SD)	23.4 (42.3)
ISUP GG	
Negative	10 (9.7)
1	4 (3.9)
2	33 (32.0)
3	22 (21.4)
4	18 (17.5)
5	16 (15.5)
Risk group (D’Amico classification)	
Negative	10 (9.7)
Low risk	2 (1.9)
Intermediate risk	38 (36.9)
High risk	53 (51.5)
N stage	
N0	83 (80.6)
N+	20 (19.4)
M stage	
M0	95 (92.2)
M1	8 (7.8)

Except where indicated, data are numbers of participants, with percentages in parentheses.

Abbreviations: ISUP GG = International Society of Urological Pathology Group Grade classification, PSA = prostate-specific antigen.

Prior to oversampling, the training set class distribution for csPCa was 63 (86.3%) ISUP GG ≥ 2 and 10 (13.7%) ISUP GG < 2 or negative; for N stage, was 16 (21.9%) N1 and 57 (78.1%) N0; and for M stage, was 6 (8.2%) M1 and 67 (91.8%) M0. The oversampled training data distribution for csPCa was 63 (51.2%) ISUP GG ≥ 2 and 60 (48.8%) ISUP GG < 2 or negative; for N stage, was 48 (45.7%) N1 and 57 (54.3%) N0; and for M Stage, was 66 (49.6%) M1 and 67 (50.4%) M0. The test data distribution for csPCa was 26 (86.7%) ISUP GG ≥ 2 and 4 (13.3%) ISUP GG < 2; for N stage, was 6 (20.0%) N1 and 24 (80.0%) N0; and for M stage, was 2 (6.7%) M1 and 28 (93.3%) M0.

Multiple models were assessed to predict csPCa via whole-gland segmentation, with the highest training accuracy and best model fit obtained from the trained full model (mean accuracy = 0.917, AIC = 68.9), showing strong associations for age (OR = 1.16, 95% CI = 1.01, 1.36, *P* = .046), PSA (OR = 1.41, 95% CI = 1.13, 1.92, *P* = .010), T2w GLZLM ZLNU (OR = 0.08, 95% CI = 0.02, 0.26, *P* < .001), ADC CONVENTIONAL Excess Kurtosis (OR = 11.1, 95% CI = 3.01, 75.7, *P* = .002), and PET DISCRETIZED SUV (OR = 0.06, 95% CI = 0.01, 0.21, *P* < .001; Table 2).

**Table 2.** ISUP GG ≥ 2 final logistic regression model estimates and accuracy from training data.

Model	Variables	OR (95% CI)	*P*-value	AIC	Accuracy, mean (range)
Clinical	Age, years	1.00 (0.94, 1.07)	.88	154	0.673 (0.417, 0.833)
	PSA, ng/mL	1.12 (1.05, 1.21)	.003		
T2w	T2 GLZLM ZLNU	0.29 (0.17, 0.48)	<.001	142	0.717 (0.417, 1.000)
ADC	ADC CONVENTIONAL ExcessKurtosis	2.78 (1.55, 5.47)	.001	150	0.653 (0.385, 1.000)
	ADC GLCM Homogeneity InverseDifference	1.80 (1.13, 2.97)	.017		
PET	PET DISCRETIZED SUVbwmin	0.19 (0.09, 0.35)	<.001	110	0.807 (0.583, 1.000)
	PET NGLDM Busyness	0.32 (0.17, 0.54)	<.001		
Clinical + T2w	Age, years	1.02 (0.95, 1.09)	.63	127	0.782 (0.583, 1.000)
	PSA, ng/mL	1.19 (1.09, 1.34)	.001		
	T2 GLZLM ZLNU	0.25 (0.13, 0.43)	<.001		
Clinival + ADC	Age, years	1.00 (0.93, 1.08)	.94	138	0.693 (0.500, 0.923)
	PSA, ng/mL	1.13 (1.05, 1.24)	.004		
	ADC CONVENTIONAL ExcessKurtosis	3.43 (1.82, 7.28)	<.001		
	ADC GLCM Homogeneity InverseDifference	1.38 (0.81, 2.37)	.24		
Clinical + PET	Age, years	1.11 (1.02, 1.23)	.023	102	0.800 (0.500, 1.000)
	PSA, ng/mL	1.08 (1.02, 1.21)	.042		
	PET DISCRETIZED SUVbwmin	0.13 (0.05, 0.28)	<.001		
	PET NGLDM Busyness	0.39 (0.20, 0.66)	.002		
T2w + ADC	T2 GLZLM ZLNU	0.19 (0.09, 0.35)	<.001	114	0.815 (0.583, 1.000)
	ADC CONVENTIONAL ExcessKurtosis	4.28 (1.96, 10.8)	.001		
	ADC GLCM Homogeneity InverseDifference	2.40 (1.38, 4.47)	.003		
T2w + PET	T2 GLZLM ZLNU	0.34 (0.17, 0.61)	.001	97.8	0.849 (0.583, 1.000)
	PET DISCRETIZED SUVbwmin	0.15 (0.06, 0.31)	<.001		
	PET NGLDM Busyness	0.41 (0.21, 0.70)	.003		
ADC + PET	ADC CONVENTIONAL ExcessKurtosis	2.85 (1.38, 6.56)	.008	104	0.812 (0.583, 1.000)
	ADC GLCM Homogeneity InverseDifference	1.20 (0.64, 2.25)	.57		
	PET DISCRETIZED SUVbwmin	0.21 (0.10, 0.39)	<.001		
	PET NGLDM Busyness	0.36 (0.19, 0.61)	.001		
T2w + ADC + PET	T2 GLZLM ZLNU	0.19 (0.07, 0.42)	<.001	84.5	0.881 (0.667, 1.000)
	ADC CONVENTIONAL ExcessKurtosis	5.54 (2.01, 19.5)	.003		
	ADC GLCM Homogeneity InverseDifference	1.38 (0.65, 3.04)	.40		
	PET DISCRETIZED SUVbwmin	0.15 (0.05, 0.33)	<.001		
	PET NGLDM Busyness	0.50 (0.24, 0.99)	.048		
Clinical + T2w + ADC	Age, years	1.01 (0.93, 1.11)	.77	98.4	0.8615 (0.583, 1.000)
	PSA, ng/mL	1.26 (1.12, 1.48)	.001		
	T2 GLZLM ZLNU	0.13 (0.05, 0.28)	<.001		
	ADC CONVENTIONAL ExcessKurtosis	6.28 (2.42, 21.7)	.001		
	ADC GLCM Homogeneity InverseDifference	2.14 (1.11, 4.49)	.031		
Clinical + T2w + PET	Age, years	1.18 (1.06, 1.36)	.008	83.9	0.8731 (0.583, 1.000)
	PSA, ng/mL	1.25 (1.06, 1.60)	.036		
	T2 GLZLM ZLNU	0.20 (0.07, 0.44)	.001		
	PET DISCRETIZED SUVbwmin	0.07 (0.02, 0.20)	<.001		
	PET NGLDM Busyness	0.49 (0.26, 0.84)	.016		
Clinical + ADC + PET	Age, years	1.11 (1.01, 1.24)	.045	94.8	0.802 (0.500, 1.000)
	PSA, ng/mL	1.10 (1.02, 1.24)	.06		
	ADC CONVENTIONAL ExcessKurtosis	3.45 (1.60, 8.58)	.003		
	ADC GLCM Homogeneity InverseDifference	0.89 (0.41, 1.83)	.75		
	PET DISCRETIZED SUVbwmin	0.15 (0.06, 0.32)	<.001		
	PET NGLDM Busyness	0.39 (0.19, 0.68)	.002		
Full	Age, years	1.16 (1.01, 1.36)	.046	68.9	0.9168 (0.6667, 1.000)
	PSA, ng/mL	1.41 (1.13, 1.92)	.010		
	T2 GLZLM ZLNU	0.08 (0.02, 0.26)	<.001		
	ADC CONVENTIONAL ExcessKurtosis	11.1 (3.01, 75.7)	.002		
	ADC GLCM Homogeneity InverseDifference	0.89 (0.32, 2.42)	.82		
	PET DISCRETIZED SUVbwmin	0.06 (0.01, 0.21)	<.001		
	PET NGLDM Busyness	0.46 (0.17, 1.03)	.07		

Accuracy from 10-fold cross-validation with 5 repeats.

Abbreviations: AIC = Akaike information criterion; CI = confidence interval; OR = odds ratio.

For models trained in the prediction of the patients’ extra-prostatic status based on M stage (M1), the highest training accuracy and good model fit was obtained from the trained full model (mean accuracy = 0.902, AIC = 86.6), showing strong associations with T2w DISCRETIZED Q2 (OR = 0.03, 95% CI = 0.00, 0.18, *P* = .001), T2w DISCRETIZED Skewness (OR = 16.9, 95% CI = 3.99, 122.0, *P* = .001), T2w DISCRETIZED HISTO ExcessKurtosis (OR = 0.12, 95% CI = 0.02, 0.64, *P* = .022), and ADC GLRLM SRE (OR = 0.10, 95% CI = 0.02, 0.30, *P* < .001) variables (Table 3).

**Table 3.** M Stage (M1) final logistic regression model estimates and accuracy from training data.

Model	Variables	OR (95% CI)	*P*-value	AIC	Accuracy, mean (range)
Clinical	Age, years	1.15 (1.08, 1.22)	<.001	165	0.651 (0.417, 0.923)
	PSA, ng/mL	1.00 (0.98, 1.02)	.91		
T2w	T2 DISCRETIZED Q2	0.17 (0.06, 0.40)	<.001	107	0.836 (0.615, 1.000)
	T2 DISCRETIZED Skewness	5.67 (2.38, 16.1)	<.001		
	T2 DISCRETIZED HISTO ExcessKurtosis	0.81 (0.29, 2.00)	.67		
ADC	ADC GLRLM SRE	0.30 (0.18, 0.47)	<.001	155	0.713 (0.429, 1.000)
Clinical + T2w	Age, years	1.05 (0.96, 1.15)	.25	109	0.819 (0.571, 1.000)
	PSA, ng/mL	0.99 (0.95, 1.02)	.44		
	T2 DISCRETIZED Q2	0.17 (0.05, 0.43)	.001		
	T2 DISCRETIZED Skewness	5.02 (2.05, 14.7)	.001		
	T2 DISCRETIZED HISTO ExcessKurtosis	0.78 (0.26, 2.03)	.63		
Clinical + ADC	Age, years	1.13 (1.05, 1.22)	.001	139	0.702 (0.429, 0.923)
	PSA, ng/mL	0.98 (0.95, 1.01)	.25		
	ADC GLRLM SRE	0.28 (0.15, 0.47)	<.001		
T2w + ADC	T2 DISCRETIZED Q2	0.04 (0.01, 0.19)	<.001	86.5	0.878 (0.692, 1.000)
	T2 DISCRETIZED Skewness	24.8 (5.29, 211.0)	.001		
	T2 DISCRETIZED HISTO ExcessKurtosis	0.12 (0.02, 0.63)	.023		
	ADC GLRLM SRE	0.12 (0.03, 0.33)	<.001		
Full	Age, years	1.01 (0.89, 1.14)	.83	86.6	0.902 (0.692, 1.000)
	PSA, ng/mL	0.97 (0.92, 1.00)	.13		
	T2 DISCRETIZED Q2	0.03 (0.00, 0.18)	.001		
	T2 DISCRETIZED Skewness	16.9 (3.99, 122.0)	.001		
	T2 DISCRETIZED HISTO ExcessKurtosis	0.12 (0.02, 0.64)	.022		
	ADC GLRLM SRE	0.10 (0.02, 0.30)	<.001		

Accuracy from 10-fold cross-validation with five repeats.

Abbreviations: AIC = Akaike information criterion; CI = confidence interval; OR = odds ratio.

Models trained in the prediction of lymph node involvement based on N stage (N1), the highest training accuracy and best model fit were from the imaging-based T2w + PET hybrid model (mean accuracy = 0.773, AIC = 99.0), demonstrating associations with T2w CONVENTIONAL Kurtosis (OR = 4.01, 95% CI = 1.06, 19.4, *P* = .06) and PET70% DISCRETIZED TLG (OR = 4.89, 95% CI = 1.02, 30.9, *P* = .07) variables (Table 4).

**Table 4.** N Stage (N1) final logistic regression model estimates and accuracy from training data.

Model	Variables	OR (95% CI)	*P*-value	AIC	Accuracy, mean (range)
Clinical	Age, years	1.03 (0.98, 1.10)	.25	140	0.620 (0.400, 0.900)
	PSA, ng/mL	1.02 (1.01, 1.04)	.023		
T2w	T2 CONVENTIONAL Skewness	1.13 (0.54, 2.39)	.75	123	0.668 (0.364, 0.909)
	T2 CONVENTIONAL Kurtosis	1.90 (0.64, 6.29)	.27		
	T2 GLRLM LRE	3.75 (1.38, 12.2)	.017		
PET	PET GLRLM LRHGE	1.52 (0.69, 5.24)	.41	115	0.727 (0.444, 1.000)
	PET70 DISCRETIZED TLG mL only for PET or NM	11.4 (2.34, 76.1)	.006		
Clinical + T2w	Age, years	1.00 (0.94, 1.08)	.89	125	0.654 (0.300, 1.000)
	PSA, ng/mL	1.01 (1.00, 1.03)	.24		
	T2 CONVENTIONAL Skewness	1.12 (0.52, 2.45)	.78		
	T2 CONVENTIONAL Kurtosis	1.80 (0.61, 5.91)	.31		
	T2 GLRLM LRE	3.38 (1.19, 11.1)	.031		
Clinical + PET	Age, years	1.03 (0.97, 1.11)	.31	117	0.721 (0.444, 1.000)
	PSA, ng/mL	1.01 (1.00, 1.02)	.32		
	PET GLRLM LRHGE	1.57 (0.73, 5.42)	.36		
	PET70 DISCRETIZED TLG mL only for PET or NM	7.76 (1.62, 54.3)	.021		
T2w + PET	T2 CONVENTIONAL Skewness	0.90 (0.37, 2.19)	.82	99.0	0.773 (0.500, 1.000)
	T2 CONVENTIONAL Kurtosis	4.01 (1.06, 19.4)	.06		
	T2 GLRLM LRE	2.17 (0.67, 8.97)	.26		
	PET GLRLM LRHGE	2.08 (1.00, 7.08)	.13		
	PET70 DISCRETIZED TLG mL only for PET or NM	4.89 (1.02, 30.9)	.07		
Full	Age, years	0.95 (0.87, 1.04)	.27	102	0.739 (0.500, 1.000)
	PSA, ng/mL	1.00 (0.98, 1.02)	.86		
	T2 CONVENTIONAL Skewness	1.02 (0.41, 2.60)	.96		
	T2 CONVENTIONAL Kurtosis	4.74 (1.17, 25.5)	.045		
	T2 GLRLM LRE	2.09 (0.56, 9.08)	.31		
	PET GLRLM LRHGE	2.04 (1.02, 6.59)	.11		
	PET70 DISCRETIZED TLG mL only for PET or NM	5.53 (1.04, 39.3)	.06		

Accuracy from 10-fold cross-validation with five repeats.

Abbreviations: AIC = Akaike information criterion; CI = confidence interval; OR = odds ratio.

Table 5 presents the comparative performance of models evaluated for the csPCa and M1 stage outcomes. Regarding the performance of models for the prediction of csPCa, the imaging-based hybrid model (T2w + PET) provided the highest AUC (0.981, 95% CI = 0.937, 1.000), followed by its age- and PSA-adjusted counterpart (AUC = 0.923, 95%CI = 0.766, 1.000) with comparable AUCs (*P* = .44). Noteworthy, for the single T2w radiomic variable (T2w GLZLM ZLNU) evaluated for the prediction of csPCa, the classification threshold based on the Youden index was 0.410 with specificity of 1.000 and sensitivity of 0.846.

**Table 5.** Sensitivity, specificity, positive and negative predictive values, and AUC of models evaluated on test data.

Outcome	Model	Sensitivity (95% CI)	Specificity (95% CI)	PPV (95% CI)	NPV (95% CI)	AUC (95% CI)
ISUP	Clinical	0.654 (0.443, 0.828)	0.500 (0.068, 0.932)	0.895 (0.669, 0.987)	0.182 (0.023, 0.518)	0.673 (0.483, 0.863)
GG ≥ 2	T2w	0.769 (0.564, 0.910)	1.000 (0.398, 1.000)	1.000 (0.832, 1.000)	0.400 (0.122, 0.738)	0.914 (0.801, 1.000)
	ADC	0.500 (0.299, 0.701)	0.500 (0.068, 0.932)	0.867 (0.595, 0.983)	0.133 (0.017, 0.405)	0.567 (0.332, 0.803)
	PET	0.885 (0.698, 0.976)	0.250 (0.006, 0.806)	0.885 (0.698, 0.976)	0.250 (0.006, 0.806)	0.664 (0.215, 1.000)
	Clinical + T2w	0.846 (0.651, 0.956)	0.750 (0.194, 0.994)	0.957 (0.781, 0.999)	0.429 (0.099, 0.816)	0.885 (0.695, 1.000)
	Clinical + ADC	0.654 (0.443, 0.828)	0.250 (0.006, 0.806)	0.850 (0.621, 0.968)	0.100 (0.003, 0.445)	0.596 (0.387, 0.806)
	Clinical + PET	0.846 (0.651, 0.956)	0.500 (0.068, 0.932)	0.917 (0.730, 0.990)	0.333 (0.043, 0.777)	0.664 (0.285, 1.000)
	T2w + ADC	0.731 (0.522, 0.884)	0.750 (0.194, 0.994)	0.950 (0.751, 0.999)	0.300 (0.067, 0.652)	0.827 (0.651, 1.000)
	T2w + PET	0.962 (0.804, 0.999)	0.500 (0.068, 0.932)	0.926 (0.757, 0.991)	0.667 (0.094, 0.992)	0.981 (0.937, 1.000)
	ADC + PET	0.885 (0.698, 0.976)	0.250 (0.006, 0.806)	0.885 (0.698, 0.976)	0.250 (0.006, 0.806)	0.577 (0.180, 0.974)
	T2w + ADC + PET	0.885 (0.698, 0.976)	0.500 (0.068, 0.932)	0.920 (0.740, 0.990)	0.400 (0.053, 0.853)	0.856 (0.659, 1.000)
	Clinical + T2w + ADC	0.808 (0.606, 0.934)	0.500 (0.068, 0.932)	0.913 (0.720, 0.989)	0.286 (0.037, 0.710)	0.846 (0.671, 1.000)
	Clinical + T2w + PET	0.923 (0.749, 0.991)	0.750 (0.194, 0.994)	0.960 (0.796, 0.999)	0.600 (0.147, 0.947)	0.923 (0.766, 1.000)
	Clinical + ADC + PET	0.846 (0.651, 0.956)	0.250 (0.006, 0.806)	0.880 (0.688, 0.975)	0.200 (0.005, 0.716)	0.615 (0.265, 0.966)
	Full	0.923 (0.749, 0.991)	0.500 (0.068, 0.932)	0.923 (0.749, 0.991)	0.500 (0.068, 0.932)	0.885 (0.730, 1.000)
M Stage	Clinical	0.000 (0.000, 0.842)	0.750 (0.551, 0.893)	0.000 (0.000, 0.410)	0.913 (0.720, 0.989)	0.500 (0.000, 1.000)
(M1)	T2w	0.500 (0.013, 0.987)	0.750 (0.551, 0.893)	0.125 (0.003, 0.527)	0.955 (0.772, 0.999)	0.786 (0.356, 1.000)
	ADC	1.000 (0.158, 1.000)	0.714 (0.513, 0.868)	0.200 (0.025, 0.556)	1.000 (0.832, 1.000)	0.875 (0.675, 1.000)
	Clinical + T2w	0.500 (0.013, 0.987)	0.821 (0.631, 0.939)	0.167 (0.004, 0.641)	0.958 (0.789, 0.999)	0.768 (0.368, 1.000)
	Clinical + ADC	0.500 (0.013, 0.987)	0.714 (0.513, 0.868)	0.111 (0.003, 0.482)	0.952 (0.762, 0.999)	0.393 (0.000, 1.000)
	T2w + ADC	0.500 (0.013, 0.987)	0.929 (0.765, 0.991)	0.333 (0.008, 0.906)	0.963 (0.810, 0.999)	0.929 (0.774, 1.000)
	Full	0.000 (0.000, 0.842)	0.893 (0.718, 0.977)	0.000 (0.000, 0.708)	0.926 (0.757, 0.991)	0.821 (0.669, 0.974)

Abbreviations: AUC = area under the receiver operating characteristic curve; CI = confidence interval; NPV = negative predictive value; PPV, positive predictive value.

The performance evaluation of N1 models showed the highest AUC for the full model of age- and PSA-adjusted T2w + PET (AUC = 0.799, 95% CI = 0.592, 1.000), followed by the hybrid T2w + PET model (AUC = 0.785, 95% CI = 0.557, 1.000), showing a similar sensitivity of 0.667 and with comparable AUCs (*P* = .57).

In terms of the performance of models for the prediction of M1 stage, the T2w + ADC model showed the highest AUC (0.929, 95% CI = 0.774, 1.000), followed by the single ADC model (AUC = 0.875, 95% CI = 0.675, 1.000) with comparable AUCs (*P* = .22). Notably, the single ADC variable (ADC GLRLM SRE) had a classification threshold of 0.556 based on the Youden index with specificity of 0.786 and sensitivity of 1.00.

## Discussion

In this study, we evaluated the association and value of whole-prostate gland-derived radiomic features in the prediction of the intra- and extra-prostatic status in 103 patients with clinically suspected or biopsy-proven PCa. Our study showed that the hybrid [^18^F]F-DCFPyL PET/MRI (T2w + PET) whole-gland-derived radiomics was the best-performing model for the detection of csPCa (AUC = 0.98). Additionally, the performance evaluation of N1 prediction models showed the highest AUC again for T2w + PET (AUC = 0.80). However, in terms of predicting the M1 stage, the MRI-only model, including both T2w and ADC, showed the highest AUC (=0.93).

There is a growing body of literature showing the value of PSMA PET radiomics derived from suspicious intraprostatic lesions. For example, Aksu et al aimed to predict the GS of the detected lesions using single-modality PSMA PET-derived radiomics features. Their developed model showed promising results in detecting patients with ISUP GG 4-5.^36^ Similarly, Zamboglu et al studied patients with intermediate and high-risk PCa and showed that radiomic features derived from individually segmented tumoral lesions could discriminate between lesions with low (ISUP GG 1-3) and high (ISUP GG 4-5) GS.^37^ Additionally, it was found that based on the fact that a higher GS was predictive of a higher chance of nodal metastasis, they were able to observe a relationship between radiomics and patients’ N staging (N0 vs N1). Cysouw et al also delineated intraprostatic tumoral lesions manually and showed that radiomic features derived from ^18^F-DCFPyL PET could predict the patients’ ISUP GG (1-3 vs 4-5; AUC = 0.81), the presence of extracapsular extension (AUC = 0.76), lymph node involvement (N0 vs N1; AUC = 0.86), and nodal/distant metastasis (AUC = 0.86).^22^

In a later study, Zamboglu et al segmented the tissue containing no visualized tumoral lesion with whole-gland contouring and found out that [^68^Ga]Ga-PSMA-11 PET-derived radiomics could identify visually missing PCa in the prostate gland.^38^ Yi et al also studied invisible intraprostatic lesions to develop and validate PSMA PET-derived radiomics models in primary PCa.^39^ The performance of their trained random forest models was calculated based on the standard PET, delayed PET, and both. In the external validation, the AUCs of the trained models were 0.90, 0.86, and 0.93 for standard PET, delayed PET, and both, respectively.

Considering the added value of MRI morphological texture in tumoral lesions, Papp et al showed that [^68^Ga]Ga-PSMA-11 PET/MRI dual-modality machine learning-based model could discriminate between the low (<4) and high (≥4) GS, with a different definition of low vs high compared, ie, to Zamboglu et al.^21^ Noteworthy, most of their high-ranked 1k fold model features were PET-derived. Similarly, Feliciani et al contoured the tumoral lesions on [^68^Ga]Ga-PSMA-11 PET/MRI images and reported the test set mean AUCs of 0.53, 0.67, and 0.49 for PET-only, MRI-only (ADC), and PET + MRI models to discriminate ISUP GG 1 from ISUP GG ≥ 2.^40^ Most recently, Basso Dias et al showed that the combined [^18^F]F-DCFPyL PET/MRI radiomic model could outperform the clinical model in PCa lesion characterization.^11^

However, contrary to our study, the above-mentioned studies segmented the tumoral lesions. Since the localization of the lesions themselves needs expertise and also can suffer from a higher interobserver variability (not necessarily within the same centre but between centres), the major difference and potential added value of our study were performing a whole-gland approach for radiomics-based prediction. This may prevent radiomics feature extraction from being significantly operator-dependent in terms of localization and contouring. Data regarding this approach (a whole-gland evaluation and not lesions themselves) are scarce. Solari et al published a comparable study to ours in terms of the intraprostatic evaluation, including T1w, T2w, and [^68^Ga]Ga-PSMA-11 PET imaging, and showed that the whole-gland evaluation could categorize patients based on their primary PCa GS (ISUP GG 1-3 vs ISUP GG 4 vs ISUP GG 5).^20^ Their best overall model (using support vector machine learning) was PET + ADC (accuracy = 0.82). Similar to our findings, they showed that even single-modality models provided a significantly accurate classification performance, outperforming the clinical parameter-based model. Notably, this study used [^68^Ga]Ga-PSMA-11, which has different resolution properties compared to the ^18^F-labeled radiopharmaceutical in our study. While, in theory, higher-resolution PET imaging should provide improved radiomic feature evaluation, there is no comparison in the literature between different PSMA tracer radiomic evaluations.

Furthermore, Ghezzo et al studied 47 PCa patients using the whole-prostate segmentation approach.^41^ Nearly half of their patients (*n* = 25) underwent [^68^Ga]Ga-PSMA-11 PET/MRI and the remainder underwent [^68^Ga]Ga-PSMA-11 PET/CT. They worked only on the PET component and reported that PET-derived radiomics combined with a machine learning approach could reach a slightly higher (not statistically significant) accuracy in predicting post-surgical ISUP GG compared to the core biopsies’ histopathological assessment. Thus, supporting the value of this highly reproducible segmentation method. However, they dichotomized their population based on ISUP GGs < 4 versus ISUP GGs 4-5, which was a different categorization from ours, thus, making their model performance not comparable to what we reached.

Although they provided acceptable predictive performances for the evaluated models, none of the previously published studies on the value of the whole-gland segmentation reviewed the value of whole-gland models in the prediction of the extra-prostatic status of the patients, which was an additional aspect of our study. Our study suggests that PSMA PET/MRI radiomics features have the potential to serve as a technique for PCa pre-biopsy risk stratification. This can be of importance in therapy selection for patients with localized disease, where different treatment options are available, including active surveillance, focal ablative therapies, and radical therapies. It also reinforces the potential of PET/MRI to be used as a one-stop shop for intraprostatic detection and distant staging of PCa, possibly providing complementary prediction through radiomics. Further prospective studies on the evaluation of incorporating PET/MRI radiomics in therapy decision-making would be required to determine whether this approach improves patient outcomes.

This study had limitations. First, we evaluated a relatively limited number of patients, which could affect the model estimates. This limitation was more prominent in the subgroup of patients with distant metastasis. Second, our study design was single-centre and no external validation was performed, limiting our results' generalizability, especially knowing that radiomics features can be sensitive to differences in vendors and protocols. However, a robust internal validation was obtained to address this issue to some extent. Third, for N and M stage categorization, a composite standard of reference was used. Albeit imperfect, we used a reference standard similar to that used in other trials,^21^^,^^40^ incorporating histopathologic correlation and, when not available, correlative imaging and clinical assessment. Notably, for intraprostatic lesions, histopathology was used as the reference standard in all participants. Lastly, we used manual segmentation for our delineation step for feature extraction. While this could be a source of significant variability in intra-organ target lesion delineation, it has been shown that there is no significant inter-reader variability in the whole-gland segmentation.^42^

In conclusion, the hybrid [^18^F]F-DCFPyL PET/MRI radiomics (whole-gland T2w + PET) was the best-performing model in our study to predict ISUP GG ≥ 2 PCa. This may indicate a potential complementary value of the whole-gland hybrid PET/MRI models for non-invasive csPCa detection. Additionally, whole-gland T2w + PET model could predict N1 disease and the T2w + ADC model showed a high accuracy for M1 prediction. Thus, our findings may suggest that assessing the prostate gland as a whole can be potentially valuable for further treatment approach personalization in PCa patients. Further studies with external validation are still required to confirm the role of whole-gland radiomics in PCa.

## Supplementary Material

tqaf014_Supplementary_Data

## Data Availability

Data generated or analysed during the study are available from the corresponding author by request.
