# Supplementary material for: [18F]F-DCFPyL PET/MRI radiomics for intraprostatic prostate cancer detection and metastases prediction using whole-gland segmentation
Source: Br J Radiol. 2025 Jan 23;98(1174):1606–14. doi: 10.1093/bjr/tqaf014 (PMC12515043; doi:10.1093/bjr/tqaf014)
Supplement: tqaf014_Supplementary_Data [file tqaf014_supplementary_data.pdf]

The British Journal of Radiology

18F-DCFPyL PET/MRI Radiomics for Intraprostatic Prostate Cancer Detection and Metastases Prediction Using Whole-Gland Segmentation

--Manuscript Draft--

Manuscript Number:	BJR-D-24-00360R3
Full Title:	18F-DCFPyL PET/MRI Radiomics for Intraprostatic Prostate Cancer Detection and Metastases Prediction Using Whole-Gland Segmentation
Short Title:	PET/MRI Radiomics for Prostate Cancer Evaluation: Whole-Gland Approach
Article Type:	Research Article
Section/Category:	Diagnostic Radiology
Keywords:	Prostate; PSMA; DCFPyL; Positron Emission Tomography; Magnetic resonance imaging; Radiomics
Corresponding Author:	Seyed Ali Mirshahvalad, MD, MPH, FEBNM<br>University Health Network<br>Toronto, CANADA
Corresponding Author Secondary Information:	
Corresponding Author's Institution:	University Health Network
Corresponding Author's Secondary Institution:	
First Author:	Seyed Ali Mirshahvalad, MD, MPH, FEBNM
First Author Secondary Information:	
Order of Authors:	Seyed Ali Mirshahvalad, MD, MPH, FEBNM
	Adriano Basso Dias
	Claudia Ortega
	Jorge Andres Abreu Gomez
	Satheesh Krishna
	Nathan Perlis
	Alejandro Berlin
	Theodorus van der Kwast
	Kartik Jhaveri
	Sangeet Ghai
	Ur Metser
	Anna Theresa Santiago
	Patrick Veit-Haibach
Order of Authors Secondary Information:	
Abstract:	Objectives<br><br>To evaluate 18F-DCFPyL-PET/MRI whole-gland-derived radiomics for detecting clinically significant (cs) prostate cancer (PCa) within the prostate gland and predicting extraprostatic metastasis (NM staging).<br><br>Methods<br><br>In this single-centre, retrospective study, therapy-naïve PCa patients who underwent

18F-DCFPyL PET/MRI were included. Whole-prostate-segmentation was performed. Feature extraction from each modality was done. The selection of potential variables was made through regularized binomial logistic regression. The oversampled training data were used to train binomial logistic regression for each outcome. The estimates of the models were calculated, and the mean accuracy was reported. The trained models were assessed on the test data for comparative evaluation of performance.

Results

A total of 103 patients (mean age=65;mean PSA=23.4) were studied. Among them, 89 had csPCa, and 20 had metastatic disease. There were 5 radiomics variables selected for the International Society of Urological Pathology Grade Group(ISUP-GG) ≥2 from T2w, ADC and PET. To detect N1, five radiomics variables were selected from the T2w and PET. For M1, four radiomics variables were selected from T2w and ADC. Regarding the performance of models for the prediction of csPCa, the imaging-based hybrid model(T2w+PET) provided the highest AUC(0.98). The performance of N1 models showed the highest AUC(0.80) for T2w+PET. To predict M1, the T2w+ADC model showed the highest AUC(0.93).

Conclusions

Whole-gland PET/MRI-radiomics may provide a reliable model to predict csPCa. Also, acceptable performance was reached for predicting metastatic disease in our limited population. Our findings may support the value of whole-gland radiomics for non-invasive csPCa detection and prediction of metastatic disease.

Powered by Editorial Manager® and ProduXion Manager® from Aries Systems Corporation

Dear Dr. Nanni,

Thanks for indicating the possibility of resubmitting our manuscript, BJR-D-24-00360R2, after a minor revision. We also thank the editorial team and distinguished reviewer for their time and suggestions. Below, you will find our responses to the reviewer's comments. We hope this time all comments are sufficiently answered and, therefore, our manuscript can be considered for publication in the British Journal of Radiology.

Kind regards

Reviewer comments

Dear Authors,
thank you for trying to respond to the comments.

My feeling is that you misunderstood my comment. My criticism on the segmentation step is not related to the delineation quality and reproducibility. My point, instead, is the radiomics feature reproducibility among the two segmentation approaches. Could you proof that the features derived from the lesion vs from the entire prostate are the same? Please, elaborate a comment.

Response: We appreciate your clarification. Actually, we would certainly not expect to be able to prove that the radiomics features derived from the lesion vs from the entire prostate are the same. *We definitively think they would be and need to be different.* The radiomic features of the entire prostate would be averaged radiomic features from the tumour lesions and the other/healthy prostate tissue. The radiomics results from the tumour lesions-only would only represent the malignant tumour features. Thus, we do not think/cannot/do not intend to prove that radiomics features derived from the lesion vs from the entire prostate are the same.

Minor point: the nomenclature of the radiofarmaceutical need to be revised according to standard format. See Coenen HH, Gee AD, Adam M, Antoni G, Cutler CS, Fujibayashi Y, Jeong JM, Mach RH, Mindt TL, Pike VW, Windhorst AD. Consensus nomenclature rules for radiopharmaceutical chemistry - Setting the record straight. Nucl Med Biol. 2017 Dec;55:v-xi. doi: 10.1016/j.nucmedbio.2017.09.004. Epub 2017 Oct 2. PMID: 29074076.

Response: Thanks for this comment. We revised the paper accordingly.

As stated at the first round of revision, the results can be considered just preliminary due to the methodological limitations that cannot be overcome (single center, retrospective, no external validation, small sample size, manual segmentations).

Response: Thank you. Again, we completely agree in this regard. In the next already planned step, we will try to address these concerns partially by opting for external validation from a

different centre. Thereafter, we will design a prospective study to make the plus points of our findings closer to our real-world daily clinic.

^{18}F-DCFPyL PET/MRI Radiomics for Intraprostatic Prostate Cancer Detection and Metastases Prediction Using Whole-Gland Segmentation

Type: Research Article

Seyed Ali Mirshahvalad, MD, MPH, FEBNM[1ϕ*], Adriano Basso Dias, MD[1ϕ], Claudia Ortega, MD[1], Jorge Andres Abreu Gomez, MD[1], Satheesh Krishna, MD[1], Nathan Perlis, MD[2], Alejandro Berlin, MD[3], Theodorus van der Kwast, MD[4], Kartik Jhaveri, MD[1], Sangeet Ghai, MD[1], Ur Metser, MD[1], Anna Theresa Santiago, MPH, MSc[5], Patrick Veit-Haibach, MD[1]

[1] Joint Department of Medical Imaging, University Medical Imaging Toronto (UMIT), University Health Network, Mount Sinai Hospital & Women's College Hospital; University of Toronto, Toronto, ON, Canada

[2] Division of Urology, Department of Surgery, Princess Margaret Cancer Centre, University Health Network, Toronto, ON, Canada.

[3] Department of Radiation Oncology, Princess Margaret Cancer Center, University Health Network & University of Toronto, Toronto, ON, Canada

[4] Laboratory Medicine Program, University Health Network, Toronto, ON, Canada

[5] Biostatistics Department, Princess Margaret Cancer Centre, University Health Network

[ϕ] These authors contributed equally to this work

***Corresponding Author:**

Seyed Ali Mirshahvalad, MD, MPH, FEBNM

Joint Department of Medical Imaging (JDMI), University Medical Imaging Toronto (UMIT), University Health Network, Mount Sinai Hospital & Women's College Hospital; University of Toronto, Toronto, ON, Canada

Tel: (+1)2893952839

E-Mail: ali.mirshahvalad@uhn.ca; mirshahvalad.sa@gmail.com

ORCID: 0000-0003-2271-9764

Acknowledgements: None

Funding: Study supported by Toronto General Toronto Western Hospital Foundation (NCT03535831) and Mount Sinai Hospital-University Health Network Academic Medical Organization Innovation Fund 2017–2019 (NCT 03149861); Toronto, ON, Canada.

Data sharing: Data generated or analyzed during the study are available from the corresponding author by request.

Conflicts of interest disclosure: PVH received travel support and IIS grants from Siemens Healthineers in the last 3 years outside of this work. Ur Metser is an advisor for POINT Biopharm.

Abstract

Objectives: To evaluate ^{18}F-DCFPyL-PET/MRI whole-gland-derived radiomics for detecting clinically significant (cs) prostate cancer (PCa) and predicting metastasis.

Methods: Therapy-naïve PCa patients who underwent ^{18}F-DCFPyL PET/MRI were included. Whole-prostate-segmentation was performed. Feature extraction from each modality was done. The selection of potential variables was made through regularized binomial logistic regression. The oversampled training data were used to train binomial logistic regression for each outcome. The estimates of the models were calculated, and the mean accuracy was reported. The trained models were assessed on the test data for comparative evaluation of performance.

Results: A total of 103 patients (mean age=65;mean PSA=23.4) were studied. Among them, 89 had csPCa, and 20 had metastatic disease. There were 5 radiomics variables selected for ISUP-GG≥2 from T2w, ADC and PET. To detect N1, five radiomics variables were selected from the T2w and PET. For M1, four radiomics variables were selected from T2w and ADC. Regarding the performance of models for the prediction of csPCa, the imaging-based hybrid model (T2w+PET) provided the highest AUC(0.98). The performance of N1 models showed the highest AUC(0.80) for T2w+PET. To predict M1, the T2w+ADC model showed the highest AUC(0.93).

Conclusions: Whole-gland PET/MRI-radiomics may provide a reliable model to predict csPCa. Also, acceptable performance was reached for predicting metastatic disease in our limited population. Our findings may support the value of whole-gland radiomics for non-invasive csPCa detection and prediction of metastatic disease.

Advances in knowledge: Whole-gland PET/MRI-radiomics, a less operator-dependent segmentation method, can be potentially used for treatment personalization in PCa patients.

Keywords: *Prostate; PSMA; DCFPyL; Positron emission tomography; Magnetic resonance imaging; Radiomics*

[^{18}F]F-DCFPyL PET/MRI Radiomics for Intraprostatic Prostate Cancer Detection and Metastases Prediction Using Whole-Gland Segmentation

Abstract

Objectives: To evaluate [^{18}F]F-DCFPyL PET/MRI whole-gland-derived radiomics for detecting clinically significant (cs) prostate cancer (PCa) within the prostate gland and predicting extraprostatic metastasis (N and M staging).

Methods: In this single-centre, retrospective study, therapy-naïve PCa patients who underwent [^{18}F]F-DCFPyL PET/MRI were included. Whole-prostate-segmentation was performed. Feature extraction from each modality was done. The selection of potential variables was made through regularized binomial logistic regression. The oversampled training data were used to train binomial logistic regression for each outcome. The estimates of the models were calculated, and the mean accuracy was reported. The trained models were assessed on the test data for comparative evaluation of performance.

Results: A total of 103 patients (mean age=65; mean PSA=23.4) were studied. Among them, 89 had csPCa, and 20 had metastatic disease. There were 5 radiomics variables selected for the International Society of Urological Pathology Grade Group (ISUP-GG) ≥2 from T2w, ADC and PET. To detect N1, five radiomics variables were selected from the T2w and PET. For M1, four radiomics variables were selected from T2w and ADC. Regarding the performance of models for the prediction of csPCa, the imaging-based hybrid model (T2w+PET) provided the highest AUC(0.98). The performance of N1 models showed the highest AUC(0.80) for T2w+PET. To predict M1, the T2w+ADC model showed the highest AUC(0.93).

Conclusions: Whole-gland PET/MRI-radiomics may provide a reliable model to predict csPCa. Also, acceptable performance was reached for predicting metastatic disease in our limited population. Our findings may support the value of whole-gland radiomics for non-invasive csPCa detection and prediction of metastatic disease.

Advances in knowledge: Whole-gland PET/MRI-radiomics, a less operator-dependent segmentation method, can be potentially used for treatment personalization in PCa patients.

Keywords: *Prostate; PSMA; DCFPyL; Positron emission tomography; Magnetic resonance imaging; Radiomics*

Trial Registration: NCT03535831. Registered 2018; NCT03149861. Registered 2017

Introduction

Prostate cancer (PCa) is the most common malignancy and the second most common cause of cancer-related mortality in the male population (1). The diagnosis is usually made by histopathologic evaluation after a targeted or systematic biopsy, most commonly performed after detecting an elevated serum prostate-specific antigen (PSA) level (2). Currently, the 5-scale International Society of Urological Pathology Grade Group (ISUP GG) is used to classify patients into different risk groups based on their Gleason score (GS) (3). This pathological group classification has clinical importance (e.g., men with ISUP GG1 mostly undergo active surveillance, while men with ISUP ≥2 often benefit from radical treatment).

Magnetic resonance imaging (MRI) is considered the imaging technique of choice for detecting clinically significant (cs) PCa (ISUP GG ≥ 2), even in biopsy-naïve patients, compared to systematic TRUS biopsies (4). Nevertheless, MRI also may miss approximately 10% of clinically significant csPCa, though there are some mixed results about how much is really missed, and some studies reported higher proportions (5, 6). Thus, further improvement of non-invasive imaging techniques is needed to improve the detection of csPCa (7).

More recently, positron emission tomography (PET) using prostate-specific membrane antigen (PSMA), an overexpressed transmembrane cell-surface protein in the PCa cells, has been extensively evaluated in different scenarios of the PCa workup (8-10). PSMA can be targeted by different PET tracers to visualize PCa lesions. [^{18}F]F-DCFPyL (2-(3-(1-carboxy-5-[(6-^{18}F-fluoro-pyridine-3-carbonyl)-amino]-pentyl)-ureido)-pentanedioic acid), a second-generation FDA-approved PSMA tracer, has demonstrated accurate results in various clinical scenarios (11-14). By merging the advantages of PSMA PET and MRI as a hybrid modality, PSMA PET/MRI can be used in the evaluation of patients with PCa (14-17).

In combination with clinical data, radiomics is a noninvasive method that has shown promising results in medical imaging, particularly in oncology (18, 19). PSMA PET/MRI-derived radiomic features have shown to be capable of improving PSMA PET/MRI baseline diagnostic accuracy (20-22). In this study, we aimed to evaluate the value of [^{18}F]F-DCFPyL PET/MRI-derived radiomic features in detecting intraprostatic csPCa and predicting nodal and distant metastases using a whole-gland approach.

Methods

Patient Data

This ethics review board-approved, retrospective analysis of two prospective clinical trials (ClinicalTrials.gov Identifiers: NCT03535831 [Cohort A] and NCT03149861 [Cohort B]) included 103 treatment-naïve men who had confirmed or suspected PCa (23). It received approval from the joint department of the medical imaging department, followed institutional guidelines and regulations, and was done in accordance with the Declaration of Helsinki. Three patient groups were recruited: patients with unfavorable intermediate or high-risk PCa (n=52), patients being considered for focal therapy (n=31), or those who had either suspicion of PCa and negative systematic biopsies or clinically discordant low-risk PCa (n=20). All patients were enrolled between June 2017 and December 2020 and underwent [^{18}F]F-DCFPyL PET/MRI. Informed consent was obtained from all participants. Exclusion criteria included patients who received prior primary therapy, PCa with significant sarcomatoid or spindle cell or neuroendocrine differentiation, or contraindication to MRI or gadolinium as per departmental safety guidelines.

Imaging Protocol

[^{18}F]F-DCFPyL was synthesized as previously described in the literature (24). PET images were acquired 118 (±20) minutes after intravenous administration of 324 (±12) MBq of [^{18}F]F-DCFPyL on the PET/MRI platform. All scans were performed using Biograph mMR (Siemens Healthcare, Germany). The imaging protocol for PET/MRI was performed as previously documented (25). In summary, whole-body MRI (top of the skull to upper thighs) was imaged with axial Dixon sequences for attenuation correction and axial VIBE T1 post-contrast. Multiparametric MRI of the prostate was done as follows: multiplanar T2, axial DWI (B-values: 0, 50, 900, 1600 s/ mm2), and T1-weighted volumetric interpolated breath-hold (VIBE) examination (15 phases, maximal temporal resolution <7 s for 1.26 min). The detailed MRI parameters are indicated in **Tables S1 and S2**. Whole-body PET was acquired with the same field of view as whole-body MRI (5– 6 bed positions; 2-3 min/bed for whole-body acquisition). An additional dedicated bed was also acquired from the pelvis.

Image Segmentation and Radiomic Features Extraction

Radiomic feature analysis of PET/MRI was obtained using LIFEx version 6.1 software (lifexsoft.org) (26) via the quantitation of various radiomics features based on the spatial arrangement and variation of pixel intensities within a defined volume of interest. Whole-prostate segmentations were performed on each modality separately (27). On MRI, whole-gland T2-weighted sequence and whole-gland apparent diffusion coefficient (ADC) map were evaluated. Since a thresholding method was not available for the MRI component (T2, ADC), manual contouring was done for the MRI-derived volumes of interest (VOIs) (by a radiologist with five years of experience in prostate MRI) in a slice-by-slice fashion to cover the entire prostate (whole-

gland segmentation). These VOIs were co-registered to the hybrid PET to contain the whole gland, and rechecked via background thresholding.

The radiomics features, including overall 307 different features (**Table S3**), were extracted from the segmented volumes in accordance with the image biomarker standardization initiative (IBSI) guidelines (28) and included the following: conventional metrics, size and shape features, and textural features. The feature extraction workflow is illustrated in **Figure 1**. This methodology was used in previous publications (29, 30).

Reference standards

Suspicious prostatic lesions were correlated with histopathology (prostate biopsy) findings in all patients. The highest reported ISUP GG was considered as the representative of each prostate gland. csPCa was defined as ISUP GG ≥2. For nodal and distant metastatic evaluation, a composite reference standard was applied to characterize all lesions identified on conventional imaging (CT and bone scan ± mpMRI) or PET and included histopathology, correlative imaging, and clinical/biochemical follow-up (**Table S4**). The prescription of ancillary imaging studies was at the discretion of the treating oncologist. For discrepant lesions, the definition of a true positive was based on the composite reference standard as assessed by 2 reviewers, with diagnostic criteria previously defined (31-34). Specifically, lymph nodes were considered positive if the typical distribution of prostate cancer metastases (nodal spread in an ascending pathway from pelvic stations to common iliac nodes and the retroperitoneum), and/or size change on follow-up congruent with interval therapy. The typical appearance of multifocal metastatic bone lesions on any modality, or concordance on two different modalities for solitary or few lesions was considered confirmatory. Bone lesions in men with undetectable PSA after radical primary

therapy only were considered benign. For suspected visceral metastases, in the absence of histological proof, correlative imaging or morphological change on follow-up imaging congruent with interval therapy was used for confirmation. Lymph nodes and skeletal and visceral lesions which did not fulfil the criteria for malignancy on both conventional imaging and PET were deemed true negative. The clinical stage for pelvic nodal (N) and distant metastases (M) following the American Joint Committee on Cancer [AJCC] Cancer Staging 8th edition were tabulated for conventional imaging and PET (35). Equivocal lesions on PET/MRI were considered negative.

Statistical analysis

Patient clinical characteristics and outcomes were summarized as means (standard deviation) or numbers (percentages). Data was split into 70% training data (n=73) and 30% test data (n=30) based on outcome. T2, ADC, and PET radiomics variables in the training data were pre-processed through standardization (centered and scaled), exclusion of zero or near-zero variance variables, and identification and exclusion of correlated predictors based on a pair-wise Pearson correlation cut-off of 0.75 indicating strong correlation. A total of 56 and 55 radiomic variables were assessed as possible predictors for ISUP GG ≥ 2, N stage and M stage, respectively (Appendix XX). The test data was centered and scaled using the pre-processing parameters of the training data. The selection of potential radiomics variables in the training data for each outcome was done through regularized binomial logistic regression using the least absolute shrinkage and selection operator (LASSO) according to the minimum lambda from 10-fold cross-validation. Imbalance in the ISUP GG ≥ 2, N stage and M stage outcomes was addressed using synthetic minority oversampling technique (SMOTE) to oversample the minority classes in the training data to yield up to 1:1 balance with the majority class. The oversampled training data were used to train binomial logistic

regression models for each outcome including a base clinical model for Age and PSA, single imaging modality (T2, ADC, or PET) models, and age- and PSA-adjusted models through 10-fold cross-validation repeated 5 times. The estimates of the odds ratios and Akaike Information Criterion (AIC) of the final models trained and the mean accuracy were reported. The trained models were assessed on the test data for comparative evaluation of performance based on sensitivity, specificity, positive predictive value, negative predictive value, and the area under the curve (AUC) of the receiver operating characteristic. The classification threshold using the Youden index for single imaging modality models with one variable was reported. Pairwise AUC comparisons were done using DeLong's test. Statistical analysis was done using the tidyverse (version 2.0.0), corrplot (version 0.92), glmnet (version 4.1-8), caret (version 6.0-94), epiR (version 2.0.70), and pROC (1.18.5) libraries in R version 4.3.1 (R Core Team, 2023).

Results

A total of 103 patients (mean age (SD) = 65.0 (8.1); mean PSA (SD) = 23.4 (42.3), **Table 1**) were studied. Based on the histopathologic evaluation, ten patients had negative results for PCa, four had ISUP GG1, and 89 men had csPCa (ISUP GG ≥2). In total, 20 and 8 patients had lymph node (N1) and distant metastasis (M1), respectively. Detailed reference standards' results for N and M staging are provided in **Table S4.** Follow-up data were available for 51 men, with a median follow-up of 18 months (range: 1-31 months). There were 5 radiomics variables selected for ISUP GG ≥2 from the T2w (T2w GLZLM ZLNU), ADC (ADC CONVENTIONAL ExcessKurtosis, ADC GLCM Homogeneity InverseDifference), and PET (PET DISCRETIZED SUV, PET NGLDM Busyness) modalities. To predict N1 disease, there were 5 radiomics variables selected from the T2w (T2 CONVENTIONAL Skewness, T2 CONVENTIONAL Kurtosis, T2 GLRLM LRE) and

PET (PET GLRLM LRHGE, PET70% DISCRETIZED TLG) modalities. For M staging prediction (M1), there were 4 radiomics variables selected from the T2w (T2 DISCRETIZED Q2, T2 DISCRETIZED Skewness, T2 DISCRETIZED HISTO ExcessKurtosis) and ADC (ADC GLRLM SRE) sequences.

Prior to oversampling, the training set class distribution for csPCa was 63 (86.3%) ISUP GG ≥2 and 10 (13.7%) ISUP GG < 2 or negative; for N stage, was 16 (21.9%) N1 and 57 (78.1%) N0; and for M stage, was 6 (8.2%) M1 and 67 (91.8%) M0. The oversampled training data distribution for csPCa was 63 (51.2%) ISUP GG ≥2 and 60 (48.8%) ISUP GG <2 or negative; for N stage, was 48 (45.7%) N1 and 57 (54.3%) N0; and for M Stage, was 66 (49.6%) M1 and 67 (50.4%) M0. The test data distribution for csPCa was 26 (86.7%) ISUP GG ≥2 and 4 (13.3%) ISUP GG <2; for N stage, was 6 (20.0%) N1 and 24 (80.0%) N0; and for M stage, was 2 (6.7%) M1 and 28 (93.3%) M0.

Multiple models were assessed to predict csPCa via whole-gland segmentation, with the highest training accuracy and best model fit obtained from the trained full model (mean accuracy = 0.917, AIC = 68.9), showing strong associations for Age (OR = 1.16, 95% CI = 1.01, 1.36, p = .046), PSA (OR = 1.41, 95% CI = 1.13, 1.92, p = .010), T2w GLZLM ZLNU (OR = 0.08, 95% CI = 0.02, 0.26, p <.001), ADC CONVENTIONAL Excess Kurtosis (OR = 11.1, 95% CI = 3.01, 75.7, p = .002), and PET DISCRETIZED SUV (OR = 0.06, 95% CI = 0.01, 0.21, p < .001; **Table 2**).

For models trained in the prediction of the patients' extra-prostatic status based on M stage (M1), the highest training accuracy and good model fit was obtained from the trained full model (mean accuracy = 0.902, AIC = 86.6), showing strong associations with T2w DISCRETIZED Q2 (OR = 0.03, 95% CI = 0.00, 0.18, p = .001), T2w DISCRETIZED Skewness (OR = 16.9, 95% CI = 3.99,

122.0, p = .001), T2w DISCRETIZED HISTO ExcessKurtosis (OR = 0.12, 95% CI = 0.02, 0.64, p = .022), and ADC GLRLM SRE (OR = 0.10, 95% CI = 0.02, 0.30, p< .001) variables (**Table 3**). Models trained in the prediction of lymph node involvement based on N stage (N1), the highest training accuracy and best model fit were from the imaging-based T2w + PET hybrid model (mean accuracy = 0.773, AIC = 99.0), demonstrating associations with T2w CONVENTIONAL Kurtosis (OR = 4.01, 95% CI = 1.06, 19.4, p = .06) and PET70% DISCRETIZED TLG (OR = 4.89, 95% CI = 1.02, 30.9, p=.07) variables (**Table 4**).

Table 5 presents the comparative performance of models evaluated for the csPCa and M1 stage outcomes. Regarding the performance of models for the prediction of csPCa, the imaging-based hybrid model (T2w + PET) provided the highest AUC (0.981, 95% CI = 0.937, 1.000), followed by its Age- and PSA-adjusted counterpart (AUC = 0.923, 95%CI = 0.766, 1.000) with comparable AUCs (p = .44). Noteworthy, for the single T2w radiomic variable (T2w GLZLM ZLNU) evaluated for the prediction of csPCa, the classification threshold based on the Youden index was 0.410 with specificity of 1.000 and sensitivity of 0.846.

The performance evaluation of N1 models showed the highest AUC for the full model of age- and PSA-adjusted T2w + PET (AUC = 0.799, 95% CI = 0.592, 1.000), followed by the hybrid T2w + PET model (AUC = 0.785, 95% CI = 0.557, 1.000), showing a similar sensitivity of 0.667 and with comparable AUCs (p = .57).

In terms of the performance of models for the prediction of M1 stage, the T2w + ADC model showed the highest AUC (0.929, 95% CI = 0.774, 1.000), followed by the single ADC model (AUC = 0.875, 95% CI = 0.675, 1.000) with comparable AUCs (p = .22). Notably, the single ADC variable (ADC GLRLM SRE) had a classification threshold of 0.556 based on the Youden index with specificity of 0.786 and sensitivity of 1.00.

Discussion

In this study, we evaluated the association and value of whole-prostate gland-derived radiomic features in the prediction of the intra- and extra-prostatic status in 103 patients with clinically suspected or biopsy-proven PCa. Our study showed that the hybrid [^{18}F]F-DCFPyL PET/MRI (T2w+PET) whole-gland-derived radiomics was the best-performing model for the detection of csPCa (AUC = 0.98). Additionally, the performance evaluation of N1 prediction models showed the highest AUC again for T2w+PET (AUC = 0.80). However, in terms of predicting the M1 stage, the MRI-only model, including both T2w and ADC, showed the highest AUC (= 0.93).

There is a growing body of literature showing the value of PSMA PET radiomics derived from suspicious intraprostatic lesions. For example, Aksu et al. aimed to predict the GS of the detected lesions using single-modality PSMA PET-derived radiomics features. Their developed model showed promising results in detecting patients with ISUP GG 4-5 (36). Similarly, Zamboglu et al. studied patients with intermediate and high-risk PCa and showed that radiomic features derived from individually segmented tumoral lesions could discriminate between lesions with low (ISUP GG 1-3) and high (ISUP GG 4-5) GS (37). Additionally, it was found that based on the fact that a higher GS was predictive of a higher chance of nodal metastasis, they were able to observe a relationship between radiomics and patients' N staging (N0 vs. N1). Cysouw et al. also delineated intra-prostatic tumoral lesions manually and showed that radiomic features derived from ^{18}F-DCFPyL PET could predict the patients' ISUP GG (1-3 vs. 4-5; AUC = 0.81), presence of extracapsular extension (AUC = 0.76), lymph node involvement (N0 vs. N1; AUC = 0.86), and nodal/distant metastasis (AUC = 0.86) (22).

In a later study, Zamboglu et al. segmented the tissue containing no visualized tumoral lesion with whole-gland contouring and found out that [^{68}Ga]Ga-PSMA-11 PET-derived radiomics could identify visually missing PCa in the prostate gland (38). Yi et al. also studied invisible intraprostatic lesions to develop and validate PSMA PET-derived radiomics models in primary PCa (39). The performance of their trained random forest models was calculated based on the standard PET, delayed PET, and both. In the external validation, the AUCs of the trained models were 0.90, 0.86, and 0.93 for standard PET, delayed PET, and both, respectively.

Considering the added value of MRI morphological texture in tumoral lesions, Papp et al. showed that [^{68}Ga]Ga-PSMA-11 PET/MRI dual-modality machine learning-based model could discriminate between the low (<4) and high (≥4) GS, with a different definition of low vs. high compared, i.e. to Zamboglu et al. (40). Noteworthy, most of their high-ranked 1k fold model features were PET-derived. Similarly, Feliciani et al. contoured the tumoral lesions on [^{68}Ga]Ga-PSMA-11 PET/MRI images and reported the test set mean AUCs of 0.53, 0.67, and 0.49 for PET-only, MRI-only (ADC), and PET+MRI models to discriminate ISUP GG 1 from ISUP GG ≥ 2 (41). Most recently, Basso Dias et al. showed that the combined [^{18}F]F-DCFPyL PET/MRI radiomic model could outperform the clinical model in prostate cancer lesion characterization (11).

However, contrary to our study, the above-mentioned studies segmented the tumoral lesions. Since the localization of the lesions themselves needs expertise and also can suffer from a higher interobserver variability (not necessarily within the same centre but between centres), the major difference and potential added value of our study were performing a whole-gland approach for radiomics-based prediction. This may prevent radiomics feature extraction from being significantly operator-dependent in terms of localization and contouring. Data regarding this

approach (a whole-gland evaluation and not lesions themselves) is scarce. Solari et al. published a comparable study to ours in terms of the intra-prostatic evaluation, including T1w, T2w, and [^{68}Ga]Ga-PSMA-11 PET imaging, and showed that the whole-gland evaluation could categorize patients based on their primary PCa GS (ISUP GG 1-3 vs. ISUP GG 4 vs. ISUP GG 5) (42). Their best overall model (using support vector machine learning) was PET+ADC (accuracy = 0.82). Similar to our findings, they showed that even single-modality models provided a significantly accurate classification performance, outperforming the clinical parameter-based model. Notably, this study used [^{68}Ga]Ga-PSMA-11, which has different resolution properties compared to the ^{18}F-labeled radiopharmaceutical in our study. While, in theory, higher-resolution PET imaging should provide improved radiomic feature evaluation, there is no comparison in the literature between different PSMA tracer radiomic evaluations.

Furthermore, Ghezzo et al. studied 47 PCa patients using the whole prostate segmentation approach (43). Nearly half of their patients (n = 25) underwent [^{68}Ga]Ga-PSMA-11 PET/MRI and the remainder underwent [^{68}Ga]Ga-PSMA-11 PET/CT. They worked only on the PET component and reported that PET-derived radiomics combined with a machine learning approach could reach a slightly higher (not statistically significant) accuracy in predicting post-surgical ISUP GG compared to the core biopsies' histopathological assessment. Thus, supporting the value of this highly reproducible segmentation method. However, they dichotomized their population based on ISUP GGs <4 versus ISUP GGs 4-5, which was a different categorization from ours, thus, making their model performance not comparable to what we reached.

Although they provided acceptable predictive performances for the evaluated models, none of the previously published studies on the value of the whole-gland segmentation reviewed the value of

whole-gland models in the prediction of the extra-prostatic status of the patients, which was an additional aspect of our study. Our study suggests that PSMA PET/MRI radiomics features have the potential to serve as a technique for PCa pre-biopsy risk stratification. This can be of importance in therapy selection for patients with localized disease, where different treatment options are available, including active surveillance, focal ablative therapies and radical therapies. It also reinforces the potential of PET/MRI to be used as a one-stop shop for intraprostatic detection and distant staging of prostate cancer, possibly providing complementary prediction through radiomics. Further prospective studies on the evaluation of incorporating PET/MRI radiomics in therapy decision-making would be required to determine whether this approach improves patient outcomes.

This study had limitations. First, we evaluated a relatively limited number of patients, which could affect the model estimates. This limitation was more prominent in the subgroup of patients with distant metastasis. Second, our study design was single-centre and no external validation was performed, limiting our results' generalizability, especially knowing that radiomics features can be sensitive to differences in vendors and protocols. However, a robust internal validation was obtained to address this issue to some extent. Third, for N and M stage categorization, a composite standard of reference was used. Albeit imperfect, we used a reference standard similar to that used in other trials [40, 41], incorporating histopathologic correlation and, when not available, correlative imaging and clinical assessment. Notably, for intraprostatic lesions, histopathology was used as the reference standard in all participants. Lastly, we used manual segmentation for our delineation step for feature extraction. While this could be a source of significant variability in intra-organ target lesion delineation, it has been shown that there is no significant inter-reader variability in the whole-gland segmentation [44].

In conclusion, the hybrid [^{18}F]F-DCFPyL PET/MRI radiomics (whole-gland T2w+PET) was the best-performing model in our study to predict ISUP GG ≥2 PCa. This may indicate a potential complementary value of the whole-gland hybrid PET/MRI models for non-invasive csPCa detection. Additionally, whole-gland T2w+PET model could predict N1 disease and the T2w+ADC model showed a high accuracy for M1 prediction. Thus, our findings may suggest that assessing the prostate gland as a whole can be potentially valuable for further treatment approach personalization in PCa patients. Further studies with external validation are still required to confirm the role of whole-gland radiomics in PCa.

References:

1. Siegel RL, Miller KD, Fuchs HE, Jemal A. Cancer statistics, 2022. CA: a cancer journal for clinicians. 2022.

2. Litwin MS, Tan H-J. The diagnosis and treatment of prostate cancer: a review. Jama. 2017; 317(24):2532-42.

3. Epstein JI, Egevad L, Amin MB, Delahunt B, Srigley JR, Humphrey PA. The 2014 International Society of Urological Pathology (ISUP) consensus conference on Gleason grading of prostatic carcinoma. The American journal of surgical pathology. 2016; 40(2):244-52.

4. Kasivisvanathan V, Rannikko AS, Borghi M, et al. MRI-targeted or standard biopsy for prostate-cancer diagnosis. New England Journal of Medicine. 2018; 378(19):1767-77.

5. Sathianathen NJ, Omer A, Harriss E, et al. Negative predictive value of multiparametric magnetic resonance imaging in the detection of clinically significant prostate cancer in the prostate imaging reporting and data system era: a systematic review and meta-analysis. Eur Urol. 2020; 78(3):402-14.

6. Schouten MG, van der Leest M, Pokorny M, et al. Why and Where do We Miss Significant Prostate Cancer with Multi-parametric Magnetic Resonance Imaging followed by Magnetic Resonance-guided and Transrectal Ultrasound-guided Biopsy in Biopsy-naïve Men? Eur Urol. 2017; 71(6):896-903.

7. Sonni I, Felker ER, Lenis AT, et al. Head-to-Head Comparison of 68Ga-PSMA-11 PET/CT and mpMRI with a Histopathology Gold Standard in the Detection, Intraprostatic Localization, and Determination of Local Extension of Primary Prostate Cancer: Results from a Prospective Single-Center Imaging Trial. J Nucl Med. 2022; 63(6):847-54.

8. Farolfi A, Calderoni L, Mattana F, et al. Current and emerging clinical applications of PSMA PET diagnostic imaging for prostate cancer. Journal of Nuclear Medicine. 2021; 62(5):596-604.

9. Wang Y, Galante JR, Haroon A, et al. The future of PSMA PET and WB MRI as next-generation imaging tools in prostate cancer. Nature Reviews Urology. 2022; 19(8):475-93.

10. Chavoshi M, Mirshahvalad SA, Metser U, Veit-Haibach P. 68Ga-PSMA PET in prostate cancer: a systematic review and meta-analysis of the observer agreement. Eur J Nucl Med Mol Imaging. 2021:1-9.

11. Basso Dias A, Finelli A, Bauman G, et al. Impact of 18F-DCFPyL PET on staging and treatment of unfavorable intermediate or high-risk prostate cancer. Radiology. 2022; 304(3):600-8.

12. Giesel FL, Will L, Lawal I, et al. Intraindividual comparison of 18F-PSMA-1007 and 18F-DCFPyL PET/CT in the prospective evaluation of patients with newly diagnosed prostate carcinoma: a pilot study. J Nucl Med. 2018; 59(7):1076-80.

13. Metser U, Zukotynski K, Mak V, et al. Effect of 18F-DCFPyL PET/CT on the management of patients with recurrent prostate cancer: results of a prospective multicenter registry trial. Radiology. 2022; 303(2):414-22.

14. Morris MJ, Rowe SP, Gorin MA, et al. Diagnostic performance of 18F-DCFPyL-PET/CT in men with biochemically recurrent prostate cancer: results from the CONDOR phase III, multicenter study. Clin Cancer Res. 2021; 27(13):3674-82.

15. Metser U, Ortega C, Perlis N, et al. Detection of clinically significant prostate cancer with 18F-DCFPyL PET/multiparametric MR. European Journal of Nuclear Medicine and Molecular Imaging. 2021; 48(11):3702-11.

16. Domachevsky L, Bernstine H, Goldberg N, Nidam M, Catalano OA, Groshar D. Comparison between pelvic PSMA-PET/MR and whole-body PSMA-PET/CT for the initial evaluation of prostate cancer: a proof of concept study. European radiology. 2020; 30(1):328-36.

17. Park SY, Zacharias C, Harrison C, et al. Gallium 68 PSMA-11 PET/MR imaging in patients with intermediate-or high-risk prostate cancer. Radiology. 2018; 288(2):495-505.

18. Lambin P, Leijenaar RT, Deist TM, et al. Radiomics: the bridge between medical imaging and personalized medicine. Nature reviews Clinical oncology. 2017; 14(12):749-62.

19. Xu M, Fang M, Zou J, et al. Using biparametric MRI radiomics signature to differentiate between benign and malignant prostate lesions. European journal of radiology. 2019; 114:38-44.

20. Solari EL, Gafita A, Schachoff S, et al. The added value of PSMA PET/MR radiomics for prostate cancer staging. European Journal of Nuclear Medicine and Molecular Imaging. 2021:1-12.

21. Papp L, Spielvogel CP, Grubmüller B, et al. Supervised machine learning enables non-invasive lesion characterization in primary prostate cancer with [68Ga]Ga-PSMA-11 PET/MRI. European Journal of Nuclear Medicine and Molecular Imaging. 2021; 48(6):1795-805.

22. Cysouw MCF, Jansen BHE, van de Brug T, et al. Machine learning-based analysis of [18F]DCFPyL PET radiomics for risk stratification in primary prostate cancer. European Journal of Nuclear Medicine and Molecular Imaging. 2021; 48(2):340-9.

23. Basso Dias A, Mirshahvalad SA, Ortega C, et al. The role of [18F]-DCFPyL PET/MRI radiomics for pathological grade group prediction in prostate cancer. Eur J Nucl Med Mol Imaging. 2023.

24. Ravert HT, Holt DP, Chen Y, et al. An improved synthesis of the radiolabeled prostate-specific membrane antigen inhibitor,[18F] DCFPyL. J Label Compd Radiopharm. 2016; 59(11):439-50.

25. Metser U, Chan R, Veit-Haibach P, Ghai S, Tau N. Comparison of MRI sequences in whole-body PET/MRI for staging of patients with high-risk prostate cancer. Am J Roentgenol. 2019; 212(2):377-81.

26. Nioche C, Orlhac F, Boughdad S, et al. LIFEx: a freeware for radiomic feature calculation in multimodality imaging to accelerate advances in the characterization of tumor heterogeneity. Cancer research. 2018; 78(16):4786-9.

27. Orlhac F, Soussan M, Maisonobe J-A, Garcia CA, Vanderlinden B, Buvat I. Tumor texture analysis in 18F-FDG PET: relationships between texture parameters, histogram indices, standardized uptake values, metabolic volumes, and total lesion glycolysis. J Nucl Med. 2014; 55(3):414-22.

28. Zwanenburg A, Vallières M, Abdalah MA, et al. The image biomarker standardization initiative: standardized quantitative radiomics for high-throughput image-based phenotyping. Radiology. 2020; 295(2):328.

29. Urraro F, Nardone VN, Reginelli A, et al. MRI Radiomics in prostate cancer: a reliability study. Frontiers in Oncology. 2021:5354.

30. Anconina R, Ortega C, Metser U, et al. Combined 18F-FDG PET/CT Radiomics and Sarcopenia Score in Predicting Relapse-Free Survival and Overall Survival in Patients With Esophagogastric Cancer. Clin Nucl Med. 2022:10.1097.

31. Joniau S, Van den Bergh L, Lerut E, et al. Mapping of pelvic lymph node metastases in prostate cancer. European urology. 2013; 63(3):450-8.

32. Zacho HD, Ravn S, Afshar-Oromieh A, Fledelius J, Ejlersen JA, Petersen LJ. Added value of 68Ga-PSMA PET/CT for the detection of bone metastases in patients with newly diagnosed prostate cancer and a previous 99mTc bone scintigraphy. EJNMMI Res. 2020; 10(1):1-9.

33. Briganti A, Suardi N, Capogrosso P, et al. Lymphatic spread of nodal metastases in high-risk prostate cancer: the ascending pathway from the pelvis to the retroperitoneum. The Prostate. 2012; 72(2):186-92.

34. Tokuda Y, Carlino LJ, Gopalan A, et al. Prostate cancer topography and patterns of lymph node metastasis. The American journal of surgical pathology. 2010; 34(12):1862.

35. Amin MB, Greene FL, Edge SB, et al. The eighth edition AJCC cancer staging manual: continuing to build a bridge from a population-based to a more "personalized" approach to cancer staging. CA: a cancer journal for clinicians. 2017; 67(2):93-9.

36. Aksu A, Vural Topuz Ö, Yılmaz G, Çapa Kaya G, Yılmaz BJAoNM. Dual time point imaging of staging PSMA PET/CT quantification; spread and radiomic analyses. 2022; 36(3):310-8.

37. Zamboglou C, Carles M, Fechter T, et al. Radiomic features from PSMA PET for non-invasive intraprostatic tumor discrimination and characterization in patients with intermediate-and high-risk prostate cancer-a comparison study with histology reference. Theranostics. 2019; 9(9):2595.

38. Zamboglou C, Bettermann AS, Gratzke C, et al. Uncovering the invisible—Prevalence, characteristics, and radiomics feature–based detection of visually undetectable intraprostatic tumor lesions in 68GaPSMA-11 PET images of patients with primary prostate cancer. European journal of nuclear medicine and molecular imaging. 2021; 48(6):1987-97.

39. Yi Z, Hu S, Lin X, et al. Machine learning-based prediction of invisible intraprostatic prostate cancer lesions on 68 Ga-PSMA-11 PET/CT in patients with primary prostate cancer. 2022; 49(5):1523-34.

40. Papp L, Spielvogel C, Grubmüller B, et al. Supervised machine learning enables non-invasive lesion characterization in primary prostate cancer with [68Ga] Ga-PSMA-11 PET/MRI. European journal of nuclear medicine and molecular imaging. 2021; 48(6):1795-805.

41. Feliciani G, Celli M, Ferroni F, et al. Radiomics Analysis on [68Ga] Ga-PSMA-11 PET and MRI-ADC for the Prediction of Prostate Cancer ISUP Grades: Preliminary Results of the BIOPSTAGE Trial. Cancers. 2022; 14(8):1888.

42. Solari EL, Gafita A, Schachoff S, et al. The added value of PSMA PET/MR radiomics for prostate cancer staging. Eur J Nucl Med Mol Imaging. 2022; 49(2):527-38.

43. Ghezzo S, Mapelli P, Bezzi C, et al. Role of [68Ga]Ga-PSMA-11 PET radiomics to predict post-surgical ISUP grade in primary prostate cancer. Eur J Nucl Med Mol Imaging. 2023; 50(8):2548-60.

44. Molière S, Hamzaoui D, Granger B, et al. Reference standard for the evaluation of automatic segmentation algorithms: Quantification of inter observer variability of manual delineation of prostate contour on MRI. Diagn Interv Imaging. 2024; 105(2):65-73.

Figures

Figure 1. Segmentation process, feature extraction, and model creation in the study.

Tables

Table 1. Patients' characteristics (n = 103).

**Characteristic**	**Value**
**Age, y, Mean (SD)**	65.0 (8.1)
**PSA level, ng/mL, Mean (SD)**	23.4 (42.3)
**ISUP GG**	
Negative	10 (9.7)
1	4 (3.9)
2	33 (32.0)
3	22 (21.4)
4	18 (17.5)
5	16 (15.5)
**Risk group (D'Amico Classification)**	
Negative	10 (9.7)
Low risk	2 (1.9)
Intermediate risk	38 (36.9)
High risk	53 (51.5)
N Stage	
N0	83 (80.6)
N+	20 (19.4)
M Stage	
M0	95 (92.2)
M1	8 (7.8)

Note. - Except where indicated, data are numbers of participants, with percentages in parentheses. PSA = prostate-specific antigen, ISUP GG = International Society of Urological Pathology Group Grade classification.

Table 2. ISUP GG ≥ 2 Final Logistic Regression Model Estimates and Accuracy from Training Data.

Model	Variables	OR (95% CI)	*P* value	AIC	Accuracy, Mean (Range)
Clinical	Age, years	1.00 (0.94, 1.07)	.88	154	0.673 (0.417, 0.833)
	PSA, ng/ml	1.12 (1.05, 1.21)	.003		
T2w	T2 GLZLM ZLNU	0.29 (0.17, 0.48)	<.001	142	0.717 (0.417, 1.000)
ADC	ADC CONVENTIONAL ExcessKurtosis	2.78 (1.55, 5.47)	.001	150	0.653 (0.385, 1.000)
	ADC GLCM Homogeneity InverseDifference	1.80 (1.13, 2.97)	.017		
PET	PET DISCRETIZED SUVbwmin	0.19 (0.09, 0.35)	<.001	110	0.807 (0.583, 1.000)
	PET NGLDM Busyness	0.32 (0.17, 0.54)	<.001		
Clinical + T2w	Age, years	1.02 (0.95, 1.09)	.63	127	0.782 (0.583, 1.000)
	PSA, ng/ml	1.19 (1.09, 1.34)	.001		
	T2 GLZLM ZLNU	0.25 (0.13, 0.43)	<.001		
Clinival + ADC	Age, years	1.00 (0.93, 1.08)	.94	138	0.693 (0.500, 0.923)
	PSA, ng/ml	1.13 (1.05, 1.24)	.004		
	ADC CONVENTIONAL ExcessKurtosis	3.43 (1.82, 7.28)	<.001		
	ADC GLCM Homogeneity InverseDifference	1.38 (0.81, 2.37)	.24		
Clinical + PET	Age, years	1.11 (1.02, 1.23)	.023	102	0.800 (0.500, 1.000)
	PSA, ng/ml	1.08 (1.02, 1.21)	.042		
	PET DISCRETIZED SUVbwmin	0.13 (0.05, 0.28)	<.001		
	PET NGLDM Busyness	0.39 (0.20, 0.66)	.002		
T2w + ADC	T2 GLZLM ZLNU	0.19 (0.09, 0.35)	<.001	114	0.815 (0.583, 1.000)
	ADC CONVENTIONAL ExcessKurtosis	4.28 (1.96, 10.8)	.001		
	ADC GLCM Homogeneity InverseDifference	2.40 (1.38, 4.47)	.003		
T2w + PET	T2 GLZLM ZLNU	0.34 (0.17, 0.61)	.001	97.8	0.849 (0.583, 1.000)
	PET DISCRETIZED SUVbwmin	0.15 (0.06, 0.31)	<.001		
	PET NGLDM Busyness	0.41 (0.21, 0.70)	.003		
ADC + PET	ADC CONVENTIONAL ExcessKurtosis	2.85 (1.38, 6.56)	.008	104	0.812 (0.583, 1.000)
	ADC GLCM Homogeneity InverseDifference	1.20 (0.64, 2.25)	.57		
	PET DISCRETIZED SUVbwmin	0.21 (0.10, 0.39)	<.001		
	PET NGLDM Busyness	0.36 (0.19, 0.61)	.001		
T2w + ADC + PET	T2 GLZLM ZLNU	0.19 (0.07, 0.42)	<.001	84.5	0.881 (0.667, 1.000)
	ADC CONVENTIONAL ExcessKurtosis	5.54 (2.01, 19.5)	.003		
	ADC GLCM Homogeneity InverseDifference	1.38 (0.65, 3.04)	.40		
	PET DISCRETIZED SUVbwmin	0.15 (0.05, 0.33)	<.001		
	PET NGLDM Busyness	0.50 (0.24, 0.99)	.048		
Clinical + T2w + ADC	Age, years	1.01 (0.93, 1.11)	.77	98.4	0.8615 (0.583, 1.000)
	PSA, ng/ml	1.26 (1.12, 1.48)	.001		
	T2 GLZLM ZLNU	0.13 (0.05, 0.28)	<.001		
	ADC CONVENTIONAL ExcessKurtosis	6.28 (2.42, 21.7)	.001		
	ADC GLCM Homogeneity InverseDifference	2.14 (1.11, 4.49)	.031		
Clinical + T2w + PET	Age, years	1.18 (1.06, 1.36)	.008	83.9	0.8731 (0.583, 1.000)
	PSA, ng/ml	1.25 (1.06, 1.60)	.036		

	T2 GLZLM ZLNU	0.20 (0.07, 0.44)	.001		
	PET DISCRETIZED SUVbwmin	0.07 (0.02, 0.20)	<.001		
	PET NGLDM Busyness	0.49 (0.26, 0.84)	.016		
Clinical + ADC + PET	Age, years	1.11 (1.01, 1.24)	.045	94.8	0.802 (0.500, 1.000)
	PSA, ng/ml	1.10 (1.02, 1.24)	.06		
	ADC CONVENTIONAL ExcessKurtosis	3.45 (1.60, 8.58)	.003		
	ADC GLCM Homogeneity InverseDifference	0.89 (0.41, 1.83)	.75		
	PET DISCRETIZED SUVbwmin	0.15 (0.06, 0.32)	<.001		
	PET NGLDM Busyness	0.39 (0.19, 0.68)	.002		
Full	Age, years	1.16 (1.01, 1.36)	.046	68.9	0.9168 (0.6667, 1.000)
	PSA, ng/ml	1.41 (1.13, 1.92)	.010		
	T2 GLZLM ZLNU	0.08 (0.02, 0.26)	<.001		
	ADC CONVENTIONAL ExcessKurtosis	11.1 (3.01, 75.7)	.002		
	ADC GLCM Homogeneity InverseDifference	0.89 (0.32, 2.42)	.82		
	PET DISCRETIZED SUVbwmin	0.06 (0.01, 0.21)	<.001		
	PET NGLDM Busyness	0.46 (0.17, 1.03)	.07		

OR, odds ratio; CI, confidence interval; AIC, Akaike Information Criterion; Accuracy from 10-fold cross-validation with 5 repeats

Table 3. M Stage (M1) Final Logistic Regression Model Estimates and Accuracy from Training Data.

Model	Variables	OR (95% CI)	*P* value	AIC	Accuracy, Mean (Range)
Clinical	Age, years	1.15 (1.08, 1.22)	<.001	165	0.651 (0.417, 0.923)
	PSA, ng/ml	1.00 (0.98, 1.02)	.91		
T2w	T2 DISCRETIZED Q2	0.17 (0.06, 0.40)	<.001	107	0.836 (0.615, 1.000)
	T2 DISCRETIZED Skewness	5.67 (2.38, 16.1)	<.001		
	T2 DISCRETIZED HISTO ExcessKurtosis	0.81 (0.29, 2.00)	.67		
ADC	ADC GLRLM SRE	0.30 (0.18, 0.47)	<.001	155	0.713 (0.429, 1.000)
Clinical + T2w	Age, years	1.05 (0.96, 1.15)	.25	109	0.819 (0.571, 1.000)
	PSA, ng/ml	0.99 (0.95, 1.02)	.44		
	T2 DISCRETIZED Q2	0.17 (0.05, 0.43)	.001		
	T2 DISCRETIZED Skewness	5.02 (2.05, 14.7)	.001		
	T2 DISCRETIZED HISTO ExcessKurtosis	0.78 (0.26, 2.03)	.63		
Clinical + ADC	Age, years	1.13 (1.05, 1.22)	.001	139	0.702 (0.429, 0.923)
	PSA, ng/ml	0.98 (0.95, 1.01)	.25		
	ADC GLRLM SRE	0.28 (0.15, 0.47)	<.001		
T2w + ADC	T2 DISCRETIZED Q2	0.04 (0.01, 0.19	<.001	86.5	0.878 (0.692, 1.000)
	T2 DISCRETIZED Skewness	24.8 (5.29, 211.0)	.001		
	T2 DISCRETIZED HISTO ExcessKurtosis	0.12 (0.02, 0.63)	.023		
	ADC GLRLM SRE	0.12 (0.03, 0.33)	<.001		
Full	Age, years	1.01 (0.89, 1.14)	.83	86.6	0.902 (0.692, 1.000)
	PSA, ng/ml	0.97 (0.92, 1.00)	.13		
	T2 DISCRETIZED Q2	0.03 (0.00, 0.18)	.001		
	T2 DISCRETIZED Skewness	16.9 (3.99, 122.0)	.001		
	T2 DISCRETIZED HISTO ExcessKurtosis	0.12 (0.02, 0.64)	.022		
	ADC GLRLM SRE	0.10 (0.02, 0.30)	<.001		

OR, odds ratio; CI, confidence interval; AIC, Akaike Information Criterion; Accuracy from 10-fold cross-validation with 5 repeats

Table 4. N Stage (N1) Final Logistic Regression Model Estimates and Accuracy from Training Data.

Model	Variables	OR (95% CI)	*P* value	AIC	Accuracy, Mean (Range)
Clinical	Age, years	1.03 (0.98, 1.10)	.25	140	0.620 (0.400, 0.900)
	PSA, ng/ml	1.02 (1.01, 1.04)	.023		
T2w	T2 CONVENTIONAL Skewness	1.13 (0.54, 2.39)	.75	123	0.668 (0.364, 0.909)
	T2 CONVENTIONAL Kurtosis	1.90 (0.64, 6.29)	.27		
	T2 GLRLM LRE	3.75 (1.38, 12.2)	.017		
PET	PET GLRLM LRHGE	1.52 (0.69, 5.24)	.41	115	0.727 (0.444, 1.000)
	PET70 DISCRETIZED TLG mL only for PET or NM	11.4 (2.34, 76.1)	.006		
Clinical + T2w	Age, years	1.00 (0.94, 1.08)	.89	125	0.654 (0.300, 1.000)
	PSA, ng/ml	1.01 (1.00, 1.03)	.24		
	T2 CONVENTIONAL Skewness	1.12 (0.52, 2.45)	.78		
	T2 CONVENTIONAL Kurtosis	1.80 (0.61, 5.91)	.31		
	T2 GLRLM LRE	3.38 (1.19, 11.1)	.031		
Clinical + PET	Age, years	1.03 (0.97, 1.11)	.31	117	0.721 (0.444, 1.000)
	PSA, ng/ml	1.01 (1.00, 1.02)	.32		
	PET GLRLM LRHGE	1.57 (0.73, 5.42)	.36		
	PET70 DISCRETIZED TLG mL only for PET or NM	7.76 (1.62, 54.3)	.021		
T2w + PET	T2 CONVENTIONAL Skewness	0.90 (0.37, 2.19)	.82	99.0	0.773 (0.500, 1.000)
	T2 CONVENTIONAL Kurtosis	4.01 (1.06, 19.4)	.06		
	T2 GLRLM LRE	2.17 (0.67, 8.97)	.26		
	PET GLRLM LRHGE	2.08 (1.00, 7.08)	.13		
	PET70 DISCRETIZED TLG mL only for PET or NM	4.89 (1.02, 30.9)	.07		
Full	Age, years	0.95 (0.87, 1.04)	.27	102	0.739 (0.500, 1.000)
	PSA, ng/ml	1.00 (0.98, 1.02)	.86		
	T2 CONVENTIONAL Skewness	1.02 (0.41, 2.60)	.96		
	T2 CONVENTIONAL Kurtosis	4.74 (1.17, 25.5)	.045		
	T2 GLRLM LRE	2.09 (0.56, 9.08)	.31		
	PET GLRLM LRHGE	2.04 (1.02, 6.59)	.11		
	PET70 DISCRETIZED TLG mL only for PET or NM	5.53 (1.04, 39.3)	.06		

OR, odds ratio; CI, confidence interval; AIC, Akaike Information Criterion; Accuracy from 10-fold cross-validation with 5 repeats

Table 5. Sensitivity, Specificity, Positive and Negative Predictive Values, and AUC of Models Evaluated on Test Data.

Outcome	Model	Sensitivity (95% CI)	Specificity (95% CI)	PPV (95% CI)	NPV (95% CI)	AUC (95% CI)
ISUP GG ≥ 2	Clinical	0.654 (0.443, 0.828)	0.500 (0.068, 0.932)	0.895 (0.669, 0.987)	0.182 (0.023, 0.518)	0.673 (0.483, 0.863)
	T2w	0.769 (0.564, 0.910)	1.000 (0.398, 1.000)	1.000 (0.832, 1.000)	0.400 (0.122, 0.738)	0.914 (0.801, 1.000)
	ADC	0.500 (0.299, 0.701)	0.500 (0.068, 0.932)	0.867 (0.595, 0.983)	0.133 (0.017, 0.405)	0.567 (0.332, 0.803)
	PET	0.885 (0.698, 0.976)	0.250 (0.006, 0.806)	0.885 (0.698, 0.976)	0.250 (0.006, 0.806)	0.664 (0.215, 1.000)
	Clinical + T2w	0.846 (0.651, 0.956)	0.750 (0.194, 0.994)	0.957 (0.781, 0.999)	0.429 (0.099, 0.816)	0.885 (0.695, 1.000)
	Clinical + ADC	0.654 (0.443, 0.828)	0.250 (0.006, 0.806)	0.850 (0.621, 0.968)	0.100 (0.003, 0.445)	0.596 (0.387, 0.806)
	Clinical + PET	0.846 (0.651, 0.956)	0.500 (0.068, 0.932)	0.917 (0.730, 0.990)	0.333 (0.043, 0.777)	0.664 (0.285, 1.000)
	T2w + ADC	0.731 (0.522, 0.884)	0.750 (0.194, 0.994)	0.950 (0.751, 0.999)	0.300 (0.067, 0.652)	0.827 (0.651, 1.000)
	T2w + PET	0.962 (0.804, 0.999)	0.500 (0.068, 0.932)	0.926 (0.757, 0.991)	0.667 (0.094, 0.992)	0.981 (0.937, 1.000)
	ADC + PET	0.885 (0.698, 0.976)	0.250 (0.006, 0.806)	0.885 (0.698, 0.976)	0.250 (0.006, 0.806)	0.577 (0.180, 0.974)
	T2w + ADC + PET	0.885 (0.698, 0.976)	0.500 (0.068, 0.932)	0.920 (0.740, 0.990)	0.400 (0.053, 0.853)	0.856 (0.659, 1.000)
	Clinical + T2w + ADC	0.808 (0.606, 0.934)	0.500 (0.068, 0.932)	0.913 (0.720, 0.989)	0.286 (0.037, 0.710)	0.846 (0.671, 1.000)
	Clinical + T2w + PET	0.923 (0.749, 0.991)	0.750 (0.194, 0.994)	0.960 (0.796, 0.999)	0.600 (0.147, 0.947)	0.923 (0.766, 1.000)
	Clinical + ADC + PET	0.846 (0.651, 0.956)	0.250 (0.006, 0.806)	0.880 (0.688, 0.975)	0.200 (0.005, 0.716)	0.615 (0.265, 0.966)
	Full	0.923 (0.749, 0.991)	0.500 (0.068, 0.932)	0.923 (0.749, 0.991)	0.500 (0.068, 0.932)	0.885 (0.730, 1.000)
M Stage (M1)	Clinical	0.000 (0.000, 0.842)	0.750 (0.551, 0.893)	0.000 (0.000, 0.410)	0.913 (0.720, 0.989)	0.500 (0.000, 1.000)
	T2w	0.500 (0.013, 0.987)	0.750 (0.551, 0.893)	0.125 (0.003, 0.527)	0.955 (0.772, 0.999)	0.786 (0.356, 1.000)
	ADC	1.000 (0.158, 1.000)	0.714 (0.513, 0.868)	0.200 (0.025, 0.556)	1.000 (0.832, 1.000)	0.875 (0.675, 1.000)
	Clinical + T2w	0.500 (0.013, 0.987)	0.821 (0.631, 0.939)	0.167 (0.004, 0.641)	0.958 (0.789, 0.999)	0.768 (0.368, 1.000)
	Clinical + ADC	0.500 (0.013, 0.987)	0.714 (0.513, 0.868)	0.111 (0.003, 0.482)	0.952 (0.762, 0.999)	0.393 (0.000, 1.000)
	T2w + ADC	0.500 (0.013, 0.987)	0.929 (0.765, 0.991)	0.333 (0.008, 0.906)	0.963 (0.810, 0.999)	0.929 (0.774, 1.000)
	Full	0.000 (0.000, 0.842)	0.893 (0.718, 0.977)	0.000 (0.000, 0.708)	0.926 (0.757, 0.991)	0.821 (0.669, 0.974)

CI, confidence interval; PPV, positive predictive value; NPV, negative predictive value; AUC, area under the receiver operating characteristic curve

[^{18}F]F-DCFPyL PET/MRI Radiomics for Intraprostatic Prostate Cancer Detection and Metastases Prediction Using Whole-Gland Segmentation

Abstract

Objectives: To evaluate [^{18}F]F-DCFPyL~~-~~ PET/MRI whole-gland-derived radiomics for detecting clinically significant (cs) prostate cancer (PCa) within the prostate gland and predicting extraprostatic metastasis (N and M staging).

Methods: In this single-centre, retrospective study, therapy-naïve PCa patients who underwent [^{18}F]F-DCFPyL ~~^{18}F-DCFPyL~~ PET/MRI were included. Whole-prostate-segmentation was performed. Feature extraction from each modality was done. The selection of potential variables was made through regularized binomial logistic regression. The oversampled training data were used to train binomial logistic regression for each outcome. The estimates of the models were calculated, and the mean accuracy was reported. The trained models were assessed on the test data for comparative evaluation of performance.

Results: A total of 103 patients (mean age=65; mean PSA=23.4) were studied. Among them, 89 had csPCa, and 20 had metastatic disease. There were 5 radiomics variables selected for the International Society of Urological Pathology Grade Group (ISUP-GG) ≥2 from T2w, ADC and PET. To detect N1, five radiomics variables were selected from the T2w and PET. For M1, four radiomics variables were selected from T2w and ADC. Regarding the performance of models for the prediction of csPCa, the imaging-based hybrid model (T2w+PET) provided the highest AUC(0.98). The performance of N1 models showed the highest AUC(0.80) for T2w+PET. To predict M1, the T2w+ADC model showed the highest AUC(0.93).

Conclusions: Whole-gland PET/MRI-radiomics may provide a reliable model to predict csPCa. Also, acceptable performance was reached for predicting metastatic disease in our limited population. Our findings may support the value of whole-gland radiomics for non-invasive csPCa detection and prediction of metastatic disease.

Advances in knowledge: Whole-gland PET/MRI-radiomics, a less operator-dependent segmentation method, can be potentially used for treatment personalization in PCa patients.

Keywords: *Prostate; PSMA; DCFPyL; Positron emission tomography; Magnetic resonance imaging; Radiomics*

Trial Registration: NCT03535831. Registered 2018; NCT03149861. Registered 2017

Introduction

Prostate cancer (PCa) is the most common malignancy and the second most common cause of cancer-related mortality in the male population (1). The diagnosis is usually made by histopathologic evaluation after a targeted or systematic biopsy, most commonly performed after detecting an elevated serum prostate-specific antigen (PSA) level (2). Currently, the 5-scale International Society of Urological Pathology Grade Group (ISUP GG) is used to classify patients into different risk groups based on their Gleason score (GS) (3). This pathological group classification has clinical importance (e.g., men with ISUP GG1 mostly undergo active surveillance, while men with ISUP ≥2 often benefit from radical treatment).

Magnetic resonance imaging (MRI) is considered the imaging technique of choice for detecting clinically significant (cs) PCa (ISUP GG ≥ 2), even in biopsy-naïve patients, compared to systematic TRUS biopsies (4). Nevertheless, MRI also may miss approximately 10% of clinically significant csPCa, though there are some mixed results about how much is really missed, and some studies reported higher proportions (5, 6). Thus, further improvement of non-invasive imaging techniques is needed to improve the detection of csPCa (7).

More recently, positron emission tomography (PET) using prostate-specific membrane antigen (PSMA), an overexpressed transmembrane cell-surface protein in the PCa cells, has been extensively evaluated in different scenarios of the PCa workup (8-10). PSMA can be targeted by different PET tracers to visualize PCa lesions. [^{18}F]F-DCFPyL ~~^{18}F-DCFPyL~~ (2-(3-(1-carboxy-5-[(6-^{18}F-fluoro-pyridine-3-carbonyl)-amino]-pentyl)-ureido)-pentanedioic acid), a second-generation FDA-approved PSMA tracer, has demonstrated accurate results in various clinical scenarios (11-14). By merging the advantages of PSMA PET and MRI as a hybrid modality, PSMA PET/MRI can be used in the evaluation of patients with PCa (14-17).

In combination with clinical data, radiomics is a noninvasive method that has shown promising results in medical imaging, particularly in oncology (18, 19). PSMA PET/MRI-derived radiomic features have shown to be capable of improving PSMA PET/MRI baseline diagnostic accuracy (20-22). In this study, we aimed to evaluate the value of [^{18}F]F-DCFPyL ~~^{18}F-DCFPyL~~ PET/MRI-derived radiomic features in detecting intraprostatic csPCa and predicting nodal and distant metastases using a whole-gland approach.

Methods

Patient Data

This ethics review board-approved, retrospective analysis of two prospective clinical trials (ClinicalTrials.gov Identifiers: NCT03535831 [Cohort A] and NCT03149861 [Cohort B]) included 103 treatment-naïve men who had confirmed or suspected PCa (23). It received approval from the joint department of the medical imaging department, followed institutional guidelines and regulations, and was done in accordance with the Declaration of Helsinki. Three patient groups were recruited: patients with unfavorable intermediate or high-risk PCa (n=52), patients being considered for focal therapy (n=31), or those who had either suspicion of PCa and negative systematic biopsies or clinically discordant low-risk PCa (n=20). All patients were enrolled between June 2017 and December 2020 and underwent [^{18}F]F-DCFPyL ~~^{18}F-DCFPyL~~ PET/MRI. Informed consent was obtained from all participants. Exclusion criteria included patients who received prior primary therapy, PCa with significant sarcomatoid or spindle cell or neuroendocrine differentiation, or contraindication to MRI or gadolinium as per departmental safety guidelines.

Imaging Protocol

[^{18}F]F-DCFPyL ~~^{18}F-DCFPyL~~ was synthesized as previously described in the literature (24). PET images were acquired 118 (±20) minutes after intravenous administration of 324 (±12) MBq of [^{18}F]F-DCFPyL ~~^{18}F-DCFPyL~~ on the PET/MRI platform. All scans were performed using Biograph mMR (Siemens Healthcare, Germany). The imaging protocol for PET/MRI was performed as previously documented (25). In summary, whole-body MRI (top of the skull to upper thighs) was imaged with axial Dixon sequences for attenuation correction and axial VIBE T1 post-contrast. Multiparametric MRI of the prostate was done as follows: multiplanar T2, axial DWI (B-values: 0, 50, 900, 1600 s/ mm2), and T1-weighted volumetric interpolated breath-hold (VIBE) examination (15 phases, maximal temporal resolution <7 s for 1.26 min). The detailed MRI parameters are indicated in **Tables S1 and S2**. Whole-body PET was acquired with the same field of view as whole-body MRI (5– 6 bed positions; 2-3 min/bed for whole-body acquisition). An additional dedicated bed was also acquired from the pelvis.

Image Segmentation and Radiomic Features Extraction

Radiomic feature analysis of PET/MRI was obtained using LIFEx version 6.1 software (lifexsoft.org) (26) via the quantitation of various radiomics features based on the spatial arrangement and variation of pixel intensities within a defined volume of interest. Whole-prostate segmentations were performed on each modality separately (27). On MRI, whole-gland T2-weighted sequence and whole-gland apparent diffusion coefficient (ADC) map were evaluated. Since a thresholding method was not available for the MRI component (T2, ADC), manual contouring was done for the MRI-derived volumes of interest (VOIs) (by a radiologist with five years of experience in prostate MRI) in a slice-by-slice fashion to cover the entire prostate (whole-

gland segmentation). These VOIs were co-registered to the hybrid PET to contain the whole gland, and rechecked via background thresholding.

The radiomics features, including overall 307 different features (**Table S3**), were extracted from the segmented volumes in accordance with the image biomarker standardization initiative (IBSI) guidelines (28) and included the following: conventional metrics, size and shape features, and textural features. The feature extraction workflow is illustrated in **Figure 1**. This methodology was used in previous publications (29, 30).

Reference standards

Suspicious prostatic lesions were correlated with histopathology (prostate biopsy) findings in all patients. The highest reported ISUP GG was considered as the representative of each prostate gland. csPCa was defined as ISUP GG ≥2. For nodal and distant metastatic evaluation, a composite reference standard was applied to characterize all lesions identified on conventional imaging (CT and bone scan ± mpMRI) or PET and included histopathology, correlative imaging, and clinical/biochemical follow-up (**Table S4**). The prescription of ancillary imaging studies was at the discretion of the treating oncologist. For discrepant lesions, the definition of a true positive was based on the composite reference standard as assessed by 2 reviewers, with diagnostic criteria previously defined (31-34). Specifically, lymph nodes were considered positive if the typical distribution of prostate cancer metastases (nodal spread in an ascending pathway from pelvic stations to common iliac nodes and the retroperitoneum), and/or size change on follow-up congruent with interval therapy. The typical appearance of multifocal metastatic bone lesions on any modality, or concordance on two different modalities for solitary or few lesions was considered confirmatory. Bone lesions in men with undetectable PSA after radical primary

therapy only were considered benign. For suspected visceral metastases, in the absence of histological proof, correlative imaging or morphological change on follow-up imaging congruent with interval therapy was used for confirmation. Lymph nodes and skeletal and visceral lesions which did not fulfil the criteria for malignancy on both conventional imaging and PET were deemed true negative. The clinical stage for pelvic nodal (N) and distant metastases (M) following the American Joint Committee on Cancer [AJCC] Cancer Staging 8th edition were tabulated for conventional imaging and PET (35). Equivocal lesions on PET/MRI were considered negative.

Statistical analysis

Patient clinical characteristics and outcomes were summarized as means (standard deviation) or numbers (percentages). Data was split into 70% training data (n=73) and 30% test data (n=30) based on outcome. T2, ADC, and PET radiomics variables in the training data were pre-processed through standardization (centered and scaled), exclusion of zero or near-zero variance variables, and identification and exclusion of correlated predictors based on a pair-wise Pearson correlation cut-off of 0.75 indicating strong correlation. A total of 56 and 55 radiomic variables were assessed as possible predictors for ISUP GG ≥ 2, N stage and M stage, respectively (Appendix XX). The test data was centered and scaled using the pre-processing parameters of the training data. The selection of potential radiomics variables in the training data for each outcome was done through regularized binomial logistic regression using the least absolute shrinkage and selection operator (LASSO) according to the minimum lambda from 10-fold cross-validation. Imbalance in the ISUP GG ≥ 2, N stage and M stage outcomes was addressed using synthetic minority oversampling technique (SMOTE) to oversample the minority classes in the training data to yield up to 1:1 balance with the majority class. The oversampled training data were used to train binomial logistic

regression models for each outcome including a base clinical model for Age and PSA, single imaging modality (T2, ADC, or PET) models, and age- and PSA-adjusted models through 10-fold cross-validation repeated 5 times. The estimates of the odds ratios and Akaike Information Criterion (AIC) of the final models trained and the mean accuracy were reported. The trained models were assessed on the test data for comparative evaluation of performance based on sensitivity, specificity, positive predictive value, negative predictive value, and the area under the curve (AUC) of the receiver operating characteristic. The classification threshold using the Youden index for single imaging modality models with one variable was reported. Pairwise AUC comparisons were done using DeLong's test. Statistical analysis was done using the tidyverse (version 2.0.0), corrplot (version 0.92), glmnet (version 4.1-8), caret (version 6.0-94), epiR (version 2.0.70), and pROC (1.18.5) libraries in R version 4.3.1 (R Core Team, 2023).

Results

A total of 103 patients (mean age (SD) = 65.0 (8.1); mean PSA (SD) = 23.4 (42.3), **Table 1**) were studied. Based on the histopathologic evaluation, ten patients had negative results for PCa, four had ISUP GG1, and 89 men had csPCa (ISUP GG ≥2). In total, 20 and 8 patients had lymph node (N1) and distant metastasis (M1), respectively. Detailed reference standards' results for N and M staging are provided in **Table S4.** Follow-up data were available for 51 men, with a median follow-up of 18 months (range: 1-31 months). There were 5 radiomics variables selected for ISUP GG ≥2 from the T2w (T2w GLZLM ZLNU), ADC (ADC CONVENTIONAL ExcessKurtosis, ADC GLCM Homogeneity InverseDifference), and PET (PET DISCRETIZED SUV, PET NGLDM Busyness) modalities. To predict N1 disease, there were 5 radiomics variables selected from the T2w (T2 CONVENTIONAL Skewness, T2 CONVENTIONAL Kurtosis, T2 GLRLM LRE) and

PET (PET GLRLM LRHGE, PET70% DISCRETIZED TLG) modalities. For M staging prediction (M1), there were 4 radiomics variables selected from the T2w (T2 DISCRETIZED Q2, T2 DISCRETIZED Skewness, T2 DISCRETIZED HISTO ExcessKurtosis) and ADC (ADC GLRLM SRE) sequences.

Prior to oversampling, the training set class distribution for csPCa was 63 (86.3%) ISUP GG ≥2 and 10 (13.7%) ISUP GG < 2 or negative; for N stage, was 16 (21.9%) N1 and 57 (78.1%) N0; and for M stage, was 6 (8.2%) M1 and 67 (91.8%) M0. The oversampled training data distribution for csPCa was 63 (51.2%) ISUP GG ≥2 and 60 (48.8%) ISUP GG <2 or negative; for N stage, was 48 (45.7%) N1 and 57 (54.3%) N0; and for M Stage, was 66 (49.6%) M1 and 67 (50.4%) M0. The test data distribution for csPCa was 26 (86.7%) ISUP GG ≥2 and 4 (13.3%) ISUP GG <2; for N stage, was 6 (20.0%) N1 and 24 (80.0%) N0; and for M stage, was 2 (6.7%) M1 and 28 (93.3%) M0.

Multiple models were assessed to predict csPCa via whole-gland segmentation, with the highest training accuracy and best model fit obtained from the trained full model (mean accuracy = 0.917, AIC = 68.9), showing strong associations for Age (OR = 1.16, 95% CI = 1.01, 1.36, p = .046), PSA (OR = 1.41, 95% CI = 1.13, 1.92, p = .010), T2w GLZLM ZLNU (OR = 0.08, 95% CI = 0.02, 0.26, p <.001), ADC CONVENTIONAL Excess Kurtosis (OR = 11.1, 95% CI = 3.01, 75.7, p = .002), and PET DISCRETIZED SUV (OR = 0.06, 95% CI = 0.01, 0.21, p < .001; **Table 2**).

For models trained in the prediction of the patients' extra-prostatic status based on M stage (M1), the highest training accuracy and good model fit was obtained from the trained full model (mean accuracy = 0.902, AIC = 86.6), showing strong associations with T2w DISCRETIZED Q2 (OR = 0.03, 95% CI = 0.00, 0.18, p = .001), T2w DISCRETIZED Skewness (OR = 16.9, 95% CI = 3.99,

122.0, p = .001), T2w DISCRETIZED HISTO ExcessKurtosis (OR = 0.12, 95% CI = 0.02, 0.64, p = .022), and ADC GLRLM SRE (OR = 0.10, 95% CI = 0.02, 0.30, p< .001) variables (**Table 3**). Models trained in the prediction of lymph node involvement based on N stage (N1), the highest training accuracy and best model fit were from the imaging-based T2w + PET hybrid model (mean accuracy = 0.773, AIC = 99.0), demonstrating associations with T2w CONVENTIONAL Kurtosis (OR = 4.01, 95% CI = 1.06, 19.4, p = .06) and PET70% DISCRETIZED TLG (OR = 4.89, 95% CI = 1.02, 30.9, p=.07) variables (**Table 4**).

Table 5 presents the comparative performance of models evaluated for the csPCa and M1 stage outcomes. Regarding the performance of models for the prediction of csPCa, the imaging-based hybrid model (T2w + PET) provided the highest AUC (0.981, 95% CI = 0.937, 1.000), followed by its Age- and PSA-adjusted counterpart (AUC = 0.923, 95%CI = 0.766, 1.000) with comparable AUCs (p = .44). Noteworthy, for the single T2w radiomic variable (T2w GLZLM ZLNU) evaluated for the prediction of csPCa, the classification threshold based on the Youden index was 0.410 with specificity of 1.000 and sensitivity of 0.846.

The performance evaluation of N1 models showed the highest AUC for the full model of age- and PSA-adjusted T2w + PET (AUC = 0.799, 95% CI = 0.592, 1.000), followed by the hybrid T2w + PET model (AUC = 0.785, 95% CI = 0.557, 1.000), showing a similar sensitivity of 0.667 and with comparable AUCs (p = .57).

In terms of the performance of models for the prediction of M1 stage, the T2w + ADC model showed the highest AUC (0.929, 95% CI = 0.774, 1.000), followed by the single ADC model (AUC = 0.875, 95% CI = 0.675, 1.000) with comparable AUCs (p = .22). Notably, the single ADC variable (ADC GLRLM SRE) had a classification threshold of 0.556 based on the Youden index with specificity of 0.786 and sensitivity of 1.00.

Discussion

In this study, we evaluated the association and value of whole-prostate gland-derived radiomic features in the prediction of the intra- and extra-prostatic status in 103 patients with clinically suspected or biopsy-proven PCa. Our study showed that the hybrid $[^{18}F]$F-DCFPyL ^{18}F-DCFPyL PET/MRI (T2w+PET) whole-gland-derived radiomics was the best-performing model for the detection of csPCa (AUC = 0.98). Additionally, the performance evaluation of N1 prediction models showed the highest AUC again for T2w+PET (AUC = 0.80). However, in terms of predicting the M1 stage, the MRI-only model, including both T2w and ADC, showed the highest AUC (= 0.93).

There is a growing body of literature showing the value of PSMA PET radiomics derived from suspicious intraprostatic lesions. For example, Aksu et al. aimed to predict the GS of the detected lesions using single-modality PSMA PET-derived radiomics features. Their developed model showed promising results in detecting patients with ISUP GG 4-5 (36). Similarly, Zamboglu et al. studied patients with intermediate and high-risk PCa and showed that radiomic features derived from individually segmented tumoral lesions could discriminate between lesions with low (ISUP GG 1-3) and high (ISUP GG 4-5) GS (37). Additionally, it was found that based on the fact that a higher GS was predictive of a higher chance of nodal metastasis, they were able to observe a relationship between radiomics and patients' N staging (N0 vs. N1). Cysouw et al. also delineated intra-prostatic tumoral lesions manually and showed that radiomic features derived from ^{18}F-DCFPyL PET could predict the patients' ISUP GG (1-3 vs. 4-5; AUC = 0.81), presence of

extracapsular extension (AUC = 0.76), lymph node involvement (N0 vs. N1; AUC = 0.86), and nodal/distant metastasis (AUC = 0.86) (22).

In a later study, Zamboglu et al. segmented the tissue containing no visualized tumoral lesion with whole-gland contouring and found out that [^{68}Ga]Ga-PSMA-11 PET-derived radiomics could identify visually missing PCa in the prostate gland (38). Yi et al. also studied invisible intraprostatic lesions to develop and validate PSMA PET-derived radiomics models in primary PCa (39). The performance of their trained random forest models was calculated based on the standard PET, delayed PET, and both. In the external validation, the AUCs of the trained models were 0.90, 0.86, and 0.93 for standard PET, delayed PET, and both, respectively.

Considering the added value of MRI morphological texture in tumoral lesions, Papp et al. showed that [^{68}Ga]Ga-PSMA-11 PET/MRI dual-modality machine learning-based model could discriminate between the low (<4) and high (≥4) GS, with a different definition of low vs. high compared, i.e. to Zamboglu et al. (40). Noteworthy, most of their high-ranked 1k fold model features were PET-derived. Similarly, Feliciani et al. contoured the tumoral lesions on [^{68}Ga]Ga-PSMA-11 PET/MRI images and reported the test set mean AUCs of 0.53, 0.67, and 0.49 for PET-only, MRI-only (ADC), and PET+MRI models to discriminate ISUP GG 1 from ISUP GG ≥ 2 (41). Most recently, Basso Dias et al. showed that the combined [^{18}F]F-DCFPyL ^{18}F-DCFPyL PET/MRI radiomic model could outperform the clinical model in prostate cancer lesion characterization (11).

However, contrary to our study, the above-mentioned studies segmented the tumoral lesions. Since the localization of the lesions themselves needs expertise and also can suffer from a higher interobserver variability (not necessarily within the same centre but between centres), the major

difference and potential added value of our study were performing a whole-gland approach for radiomics-based prediction. This may prevent radiomics feature extraction from being significantly operator-dependent in terms of localization and contouring. Data regarding this approach (a whole-gland evaluation and not lesions themselves) is scarce. Solari et al. published a comparable study to ours in terms of the intra-prostatic evaluation, including T1w, T2w, and [^{68}Ga]Ga-PSMA-11 PET imaging, and showed that the whole-gland evaluation could categorize patients based on their primary PCa GS (ISUP GG 1-3 vs. ISUP GG 4 vs. ISUP GG 5) (42). Their best overall model (using support vector machine learning) was PET+ADC (accuracy = 0.82). Similar to our findings, they showed that even single-modality models provided a significantly accurate classification performance, outperforming the clinical parameter-based model. Notably, this study used [^{68}Ga]Ga-PSMA-11, which has different resolution properties compared to the ^{18}F-labeled radiopharmaceutical in our study. While, in theory, higher-resolution PET imaging should provide improved radiomic feature evaluation, there is no comparison in the literature between different PSMA tracer radiomic evaluations.

Furthermore, Ghezzo et al. studied 47 PCa patients using the whole prostate segmentation approach (43). Nearly half of their patients (n = 25) underwent [^{68}Ga]Ga-PSMA-11 PET/MRI and the remainder underwent [^{68}Ga]Ga-PSMA-11 PET/CT. They worked only on the PET component and reported that PET-derived radiomics combined with a machine learning approach could reach a slightly higher (not statistically significant) accuracy in predicting post-surgical ISUP GG compared to the core biopsies' histopathological assessment. Thus, supporting the value of this highly reproducible segmentation method. However, they dichotomized their population based on ISUP GGs <4 versus ISUP GGs 4-5, which was a different categorization from ours, thus, making their model performance not comparable to what we reached.

Although they provided acceptable predictive performances for the evaluated models, none of the previously published studies on the value of the whole-gland segmentation reviewed the value of whole-gland models in the prediction of the extra-prostatic status of the patients, which was an additional aspect of our study. Our study suggests that PSMA PET/MRI radiomics features have the potential to serve as a technique for PCa pre-biopsy risk stratification. This can be of importance in therapy selection for patients with localized disease, where different treatment options are available, including active surveillance, focal ablative therapies and radical therapies. It also reinforces the potential of PET/MRI to be used as a one-stop shop for intraprostatic detection and distant staging of prostate cancer, possibly providing complementary prediction through radiomics. Further prospective studies on the evaluation of incorporating PET/MRI radiomics in therapy decision-making would be required to determine whether this approach improves patient outcomes.

This study had limitations. First, we evaluated a relatively limited number of patients, which could affect the model estimates. This limitation was more prominent in the subgroup of patients with distant metastasis. Second, our study design was single-centre and no external validation was performed, limiting our results' generalizability, especially knowing that radiomics features can be sensitive to differences in vendors and protocols. However, a robust internal validation was obtained to address this issue to some extent. Third, for N and M stage categorization, a composite standard of reference was used. Albeit imperfect, we used a reference standard similar to that used in other trials [40, 41], incorporating histopathologic correlation and, when not available, correlative imaging and clinical assessment. Notably, for intraprostatic lesions, histopathology was used as the reference standard in all participants. Lastly, we used manual segmentation for our delineation step for feature extraction. While this could be a source of significant variability in

intra-organ target lesion delineation, it has been shown that there is no significant inter-reader variability in the whole-gland segmentation [44].

In conclusion, the hybrid [^{18}F]F-DCFPyL ^{18}F-DCFPyL PET/MRI radiomics (whole-gland T2w+PET) was the best-performing model in our study to predict ISUP GG ≥2 PCa. This may indicate a potential complementary value of the whole-gland hybrid PET/MRI models for non-invasive csPCa detection. Additionally, whole-gland T2w+PET model could predict N1 disease and the T2w+ADC model showed a high accuracy for M1 prediction. Thus, our findings may suggest that assessing the prostate gland as a whole can be potentially valuable for further treatment approach personalization in PCa patients. Further studies with external validation are still required to confirm the role of whole-gland radiomics in PCa.

References:

1. Siegel RL, Miller KD, Fuchs HE, Jemal A. Cancer statistics, 2022. CA: a cancer journal for clinicians. 2022.

2. Litwin MS, Tan H-J. The diagnosis and treatment of prostate cancer: a review. Jama. 2017; 317(24):2532-42.

3. Epstein JI, Egevad L, Amin MB, Delahunt B, Srigley JR, Humphrey PA. The 2014 International Society of Urological Pathology (ISUP) consensus conference on Gleason grading of prostatic carcinoma. The American journal of surgical pathology. 2016; 40(2):244-52.

4. Kasivisvanathan V, Rannikko AS, Borghi M, et al. MRI-targeted or standard biopsy for prostate-cancer diagnosis. New England Journal of Medicine. 2018; 378(19):1767-77.

5. Sathianathen NJ, Omer A, Harriss E, et al. Negative predictive value of multiparametric magnetic resonance imaging in the detection of clinically significant prostate cancer in the prostate imaging reporting and data system era: a systematic review and meta-analysis. Eur Urol. 2020; 78(3):402-14.

6. Schouten MG, van der Leest M, Pokorny M, et al. Why and Where do We Miss Significant Prostate Cancer with Multi-parametric Magnetic Resonance Imaging followed by Magnetic Resonance-guided and Transrectal Ultrasound-guided Biopsy in Biopsy-naïve Men? Eur Urol. 2017; 71(6):896-903.

7. Sonni I, Felker ER, Lenis AT, et al. Head-to-Head Comparison of 68Ga-PSMA-11 PET/CT and mpMRI with a Histopathology Gold Standard in the Detection, Intraprostatic Localization, and Determination of Local Extension of Primary Prostate Cancer: Results from a Prospective Single-Center Imaging Trial. J Nucl Med. 2022; 63(6):847-54.

8. Farolfi A, Calderoni L, Mattana F, et al. Current and emerging clinical applications of PSMA PET diagnostic imaging for prostate cancer. Journal of Nuclear Medicine. 2021; 62(5):596-604.

9. Wang Y, Galante JR, Haroon A, et al. The future of PSMA PET and WB MRI as next-generation imaging tools in prostate cancer. Nature Reviews Urology. 2022; 19(8):475-93.

10. Chavoshi M, Mirshahvalad SA, Metser U, Veit-Haibach P. 68Ga-PSMA PET in prostate cancer: a systematic review and meta-analysis of the observer agreement. Eur J Nucl Med Mol Imaging. 2021:1-9.

11. Basso Dias A, Finelli A, Bauman G, et al. Impact of 18F-DCFPyL PET on staging and treatment of unfavorable intermediate or high-risk prostate cancer. Radiology. 2022; 304(3):600-8.

12. Giesel FL, Will L, Lawal I, et al. Intraindividual comparison of 18F-PSMA-1007 and 18F-DCFPyL PET/CT in the prospective evaluation of patients with newly diagnosed prostate carcinoma: a pilot study. J Nucl Med. 2018; 59(7):1076-80.

13. Metser U, Zukotynski K, Mak V, et al. Effect of 18F-DCFPyL PET/CT on the management of patients with recurrent prostate cancer: results of a prospective multicenter registry trial. Radiology. 2022; 303(2):414-22.

14. Morris MJ, Rowe SP, Gorin MA, et al. Diagnostic performance of 18F-DCFPyL-PET/CT in men with biochemically recurrent prostate cancer: results from the CONDOR phase III, multicenter study. Clin Cancer Res. 2021; 27(13):3674-82.

15. Metser U, Ortega C, Perlis N, et al. Detection of clinically significant prostate cancer with 18F-DCFPyL PET/multiparametric MR. European Journal of Nuclear Medicine and Molecular Imaging. 2021; 48(11):3702-11.

16. Domachevsky L, Bernstine H, Goldberg N, Nidam M, Catalano OA, Groshar D. Comparison between pelvic PSMA-PET/MR and whole-body PSMA-PET/CT for the initial evaluation of prostate cancer: a proof of concept study. European radiology. 2020; 30(1):328-36.

17. Park SY, Zacharias C, Harrison C, et al. Gallium 68 PSMA-11 PET/MR imaging in patients with intermediate-or high-risk prostate cancer. Radiology. 2018; 288(2):495-505.

18. Lambin P, Leijenaar RT, Deist TM, et al. Radiomics: the bridge between medical imaging and personalized medicine. Nature reviews Clinical oncology. 2017; 14(12):749-62.

19. Xu M, Fang M, Zou J, et al. Using biparametric MRI radiomics signature to differentiate between benign and malignant prostate lesions. European journal of radiology. 2019; 114:38-44.

20. Solari EL, Gafita A, Schachoff S, et al. The added value of PSMA PET/MR radiomics for prostate cancer staging. European Journal of Nuclear Medicine and Molecular Imaging. 2021:1-12.

21. Papp L, Spielvogel CP, Grubmüller B, et al. Supervised machine learning enables non-invasive lesion characterization in primary prostate cancer with [68Ga]Ga-PSMA-11 PET/MRI. European Journal of Nuclear Medicine and Molecular Imaging. 2021; 48(6):1795-805.

22. Cysouw MCF, Jansen BHE, van de Brug T, et al. Machine learning-based analysis of [18F]DCFPyL PET radiomics for risk stratification in primary prostate cancer. European Journal of Nuclear Medicine and Molecular Imaging. 2021; 48(2):340-9.

23. Basso Dias A, Mirshahvalad SA, Ortega C, et al. The role of [18F]-DCFPyL PET/MRI radiomics for pathological grade group prediction in prostate cancer. Eur J Nucl Med Mol Imaging. 2023.

24. Ravert HT, Holt DP, Chen Y, et al. An improved synthesis of the radiolabeled prostate-specific membrane antigen inhibitor,[18F] DCFPyL. J Label Compd Radiopharm. 2016; 59(11):439-50.

25. Metser U, Chan R, Veit-Haibach P, Ghai S, Tau N. Comparison of MRI sequences in whole-body PET/MRI for staging of patients with high-risk prostate cancer. Am J Roentgenol. 2019; 212(2):377-81.

26. Nioche C, Orlhac F, Boughdad S, et al. LIFEx: a freeware for radiomic feature calculation in multimodality imaging to accelerate advances in the characterization of tumor heterogeneity. Cancer research. 2018; 78(16):4786-9.

27. Orlhac F, Soussan M, Maisonobe J-A, Garcia CA, Vanderlinden B, Buvat I. Tumor texture analysis in 18F-FDG PET: relationships between texture parameters, histogram indices, standardized uptake values, metabolic volumes, and total lesion glycolysis. J Nucl Med. 2014; 55(3):414-22.

28. Zwanenburg A, Vallières M, Abdalah MA, et al. The image biomarker standardization initiative: standardized quantitative radiomics for high-throughput image-based phenotyping. Radiology. 2020; 295(2):328.

29. Urraro F, Nardone VN, Reginelli A, et al. MRI Radiomics in prostate cancer: a reliability study. Frontiers in Oncology. 2021:5354.

30. Anconina R, Ortega C, Metser U, et al. Combined 18F-FDG PET/CT Radiomics and Sarcopenia Score in Predicting Relapse-Free Survival and Overall Survival in Patients With Esophagogastric Cancer. Clin Nucl Med. 2022:10.1097.

31. Joniau S, Van den Bergh L, Lerut E, et al. Mapping of pelvic lymph node metastases in prostate cancer. European urology. 2013; 63(3):450-8.

32. Zacho HD, Ravn S, Afshar-Oromieh A, Fledelius J, Ejlersen JA, Petersen LJ. Added value of 68Ga-PSMA PET/CT for the detection of bone metastases in patients with newly diagnosed prostate cancer and a previous 99mTc bone scintigraphy. EJNMMI Res. 2020; 10(1):1-9.

33. Briganti A, Suardi N, Capogrosso P, et al. Lymphatic spread of nodal metastases in high-risk prostate cancer: the ascending pathway from the pelvis to the retroperitoneum. The Prostate. 2012; 72(2):186-92.

34. Tokuda Y, Carlino LJ, Gopalan A, et al. Prostate cancer topography and patterns of lymph node metastasis. The American journal of surgical pathology. 2010; 34(12):1862.

35. Amin MB, Greene FL, Edge SB, et al. The eighth edition AJCC cancer staging manual: continuing to build a bridge from a population-based to a more "personalized" approach to cancer staging. CA: a cancer journal for clinicians. 2017; 67(2):93-9.

36. Aksu A, Vural Topuz Ö, Yılmaz G, Çapa Kaya G, Yılmaz BJAoNM. Dual time point imaging of staging PSMA PET/CT quantification; spread and radiomic analyses. 2022; 36(3):310-8.

37. Zamboglou C, Carles M, Fechter T, et al. Radiomic features from PSMA PET for non-invasive intraprostatic tumor discrimination and characterization in patients with intermediate-and high-risk prostate cancer-a comparison study with histology reference. Theranostics. 2019; 9(9):2595.

38. Zamboglou C, Bettermann AS, Gratzke C, et al. Uncovering the invisible—Prevalence, characteristics, and radiomics feature–based detection of visually undetectable intraprostatic tumor lesions in 68GaPSMA-11 PET images of patients with primary prostate cancer. European journal of nuclear medicine and molecular imaging. 2021; 48(6):1987-97.

39. Yi Z, Hu S, Lin X, et al. Machine learning-based prediction of invisible intraprostatic prostate cancer lesions on 68 Ga-PSMA-11 PET/CT in patients with primary prostate cancer. 2022; 49(5):1523-34.

40. Papp L, Spielvogel C, Grubmüller B, et al. Supervised machine learning enables non-invasive lesion characterization in primary prostate cancer with [68Ga] Ga-PSMA-11 PET/MRI. European journal of nuclear medicine and molecular imaging. 2021; 48(6):1795-805.

41. Feliciani G, Celli M, Ferroni F, et al. Radiomics Analysis on [68Ga] Ga-PSMA-11 PET and MRI-ADC for the Prediction of Prostate Cancer ISUP Grades: Preliminary Results of the BIOPSTAGE Trial. Cancers. 2022; 14(8):1888.

42. Solari EL, Gafita A, Schachoff S, et al. The added value of PSMA PET/MR radiomics for prostate cancer staging. Eur J Nucl Med Mol Imaging. 2022; 49(2):527-38.

43. Ghezzo S, Mapelli P, Bezzi C, et al. Role of [68Ga]Ga-PSMA-11 PET radiomics to predict post-surgical ISUP grade in primary prostate cancer. Eur J Nucl Med Mol Imaging. 2023; 50(8):2548-60.

44. Molière S, Hamzaoui D, Granger B, et al. Reference standard for the evaluation of automatic segmentation algorithms: Quantification of inter observer variability of manual delineation of prostate contour on MRI. Diagn Interv Imaging. 2024; 105(2):65-73.

Figures

Figure 1. Segmentation process, feature extraction, and model creation in the study.

Tables

Table 1. Patients' characteristics (n = 103).

**Characteristic**	**Value**
**Age, y, Mean (SD)**	65.0 (8.1)
**PSA level, ng/mL, Mean (SD)**	23.4 (42.3)
**ISUP GG**	
Negative	10 (9.7)
1	4 (3.9)
2	33 (32.0)
3	22 (21.4)
4	18 (17.5)
5	16 (15.5)
**Risk group (D'Amico Classification)**	
Negative	10 (9.7)
Low risk	2 (1.9)
Intermediate risk	38 (36.9)
High risk	53 (51.5)
N Stage	
N0	83 (80.6)
N+	20 (19.4)
M Stage	
M0	95 (92.2)
M1	8 (7.8)

Note. - Except where indicated, data are numbers of participants, with percentages in parentheses. PSA = prostate-specific antigen, ISUP GG = International Society of Urological Pathology Group Grade classification.

Table 2. ISUP GG ≥ 2 Final Logistic Regression Model Estimates and Accuracy from Training Data.

Model	Variables	OR (95% CI)	*P* value	AIC	Accuracy, Mean (Range)
Clinical	Age, years	1.00 (0.94, 1.07)	.88	154	0.673 (0.417, 0.833)
	PSA, ng/ml	1.12 (1.05, 1.21)	.003		
T2w	T2 GLZLM ZLNU	0.29 (0.17, 0.48)	<.001	142	0.717 (0.417, 1.000)
ADC	ADC CONVENTIONAL ExcessKurtosis	2.78 (1.55, 5.47)	.001	150	0.653 (0.385, 1.000)
	ADC GLCM Homogeneity InverseDifference	1.80 (1.13, 2.97)	.017		
PET	PET DISCRETIZED SUVbwmin	0.19 (0.09, 0.35)	<.001	110	0.807 (0.583, 1.000)
	PET NGLDM Busyness	0.32 (0.17, 0.54)	<.001		
Clinical + T2w	Age, years	1.02 (0.95, 1.09)	.63	127	0.782 (0.583, 1.000)
	PSA, ng/ml	1.19 (1.09, 1.34)	.001		
	T2 GLZLM ZLNU	0.25 (0.13, 0.43)	<.001		
Clinival + ADC	Age, years	1.00 (0.93, 1.08)	.94	138	0.693 (0.500, 0.923)
	PSA, ng/ml	1.13 (1.05, 1.24)	.004		
	ADC CONVENTIONAL ExcessKurtosis	3.43 (1.82, 7.28)	<.001		
	ADC GLCM Homogeneity InverseDifference	1.38 (0.81, 2.37)	.24		
Clinical + PET	Age, years	1.11 (1.02, 1.23)	.023	102	0.800 (0.500, 1.000)
	PSA, ng/ml	1.08 (1.02, 1.21)	.042		
	PET DISCRETIZED SUVbwmin	0.13 (0.05, 0.28)	<.001		
	PET NGLDM Busyness	0.39 (0.20, 0.66)	.002		
T2w + ADC	T2 GLZLM ZLNU	0.19 (0.09, 0.35)	<.001	114	0.815 (0.583, 1.000)
	ADC CONVENTIONAL ExcessKurtosis	4.28 (1.96, 10.8)	.001		
	ADC GLCM Homogeneity InverseDifference	2.40 (1.38, 4.47)	.003		
T2w + PET	T2 GLZLM ZLNU	0.34 (0.17, 0.61)	.001	97.8	0.849 (0.583, 1.000)
	PET DISCRETIZED SUVbwmin	0.15 (0.06, 0.31)	<.001		
	PET NGLDM Busyness	0.41 (0.21, 0.70)	.003		
ADC + PET	ADC CONVENTIONAL ExcessKurtosis	2.85 (1.38, 6.56)	.008	104	0.812 (0.583, 1.000)
	ADC GLCM Homogeneity InverseDifference	1.20 (0.64, 2.25)	.57		
	PET DISCRETIZED SUVbwmin	0.21 (0.10, 0.39)	<.001		
	PET NGLDM Busyness	0.36 (0.19, 0.61)	.001		
T2w + ADC + PET	T2 GLZLM ZLNU	0.19 (0.07, 0.42)	<.001	84.5	0.881 (0.667, 1.000)
	ADC CONVENTIONAL ExcessKurtosis	5.54 (2.01, 19.5)	.003		
	ADC GLCM Homogeneity InverseDifference	1.38 (0.65, 3.04)	.40		
	PET DISCRETIZED SUVbwmin	0.15 (0.05, 0.33)	<.001		
	PET NGLDM Busyness	0.50 (0.24, 0.99)	.048		
Clinical + T2w + ADC	Age, years	1.01 (0.93, 1.11)	.77	98.4	0.8615 (0.583, 1.000)
	PSA, ng/ml	1.26 (1.12, 1.48)	.001		
	T2 GLZLM ZLNU	0.13 (0.05, 0.28)	<.001		
	ADC CONVENTIONAL ExcessKurtosis	6.28 (2.42, 21.7)	.001		
	ADC GLCM Homogeneity InverseDifference	2.14 (1.11, 4.49)	.031		
Clinical + T2w + PET	Age, years	1.18 (1.06, 1.36)	.008	83.9	0.8731 (0.583, 1.000)
	PSA, ng/ml	1.25 (1.06, 1.60)	.036		

	T2 GLZLM ZLNU	0.20 (0.07, 0.44)	.001		
	PET DISCRETIZED SUVbwmin	0.07 (0.02, 0.20)	<.001		
	PET NGLDM Busyness	0.49 (0.26, 0.84)	.016		
Clinical + ADC + PET	Age, years	1.11 (1.01, 1.24)	.045	94.8	0.802 (0.500, 1.000)
	PSA, ng/ml	1.10 (1.02, 1.24)	.06		
	ADC CONVENTIONAL ExcessKurtosis	3.45 (1.60, 8.58)	.003		
	ADC GLCM Homogeneity InverseDifference	0.89 (0.41, 1.83)	.75		
	PET DISCRETIZED SUVbwmin	0.15 (0.06, 0.32)	<.001		
	PET NGLDM Busyness	0.39 (0.19, 0.68)	.002		
Full	Age, years	1.16 (1.01, 1.36)	.046	68.9	0.9168 (0.6667, 1.000)
	PSA, ng/ml	1.41 (1.13, 1.92)	.010		
	T2 GLZLM ZLNU	0.08 (0.02, 0.26)	<.001		
	ADC CONVENTIONAL ExcessKurtosis	11.1 (3.01, 75.7)	.002		
	ADC GLCM Homogeneity InverseDifference	0.89 (0.32, 2.42)	.82		
	PET DISCRETIZED SUVbwmin	0.06 (0.01, 0.21)	<.001		
	PET NGLDM Busyness	0.46 (0.17, 1.03)	.07		

OR, odds ratio; CI, confidence interval; AIC, Akaike Information Criterion; Accuracy from 10-fold cross-validation with 5 repeats

Table 3. M Stage (M1) Final Logistic Regression Model Estimates and Accuracy from Training Data.

Model	Variables	OR (95% CI)	*P* value	AIC	Accuracy, Mean (Range)
Clinical	Age, years	1.15 (1.08, 1.22)	<.001	165	0.651 (0.417, 0.923)
	PSA, ng/ml	1.00 (0.98, 1.02)	.91		
T2w	T2 DISCRETIZED Q2	0.17 (0.06, 0.40)	<.001	107	0.836 (0.615, 1.000)
	T2 DISCRETIZED Skewness	5.67 (2.38, 16.1)	<.001		
	T2 DISCRETIZED HISTO ExcessKurtosis	0.81 (0.29, 2.00)	.67		
ADC	ADC GLRLM SRE	0.30 (0.18, 0.47)	<.001	155	0.713 (0.429, 1.000)
Clinical + T2w	Age, years	1.05 (0.96, 1.15)	.25	109	0.819 (0.571, 1.000)
	PSA, ng/ml	0.99 (0.95, 1.02)	.44		
	T2 DISCRETIZED Q2	0.17 (0.05, 0.43)	.001		
	T2 DISCRETIZED Skewness	5.02 (2.05, 14.7)	.001		
	T2 DISCRETIZED HISTO ExcessKurtosis	0.78 (0.26, 2.03)	.63		
Clinical + ADC	Age, years	1.13 (1.05, 1.22)	.001	139	0.702 (0.429, 0.923)
	PSA, ng/ml	0.98 (0.95, 1.01)	.25		
	ADC GLRLM SRE	0.28 (0.15, 0.47)	<.001		
T2w + ADC	T2 DISCRETIZED Q2	0.04 (0.01, 0.19	<.001	86.5	0.878 (0.692, 1.000)
	T2 DISCRETIZED Skewness	24.8 (5.29, 211.0)	.001		
	T2 DISCRETIZED HISTO ExcessKurtosis	0.12 (0.02, 0.63)	.023		
	ADC GLRLM SRE	0.12 (0.03, 0.33)	<.001		
Full	Age, years	1.01 (0.89, 1.14)	.83	86.6	0.902 (0.692, 1.000)
	PSA, ng/ml	0.97 (0.92, 1.00)	.13		
	T2 DISCRETIZED Q2	0.03 (0.00, 0.18)	.001		
	T2 DISCRETIZED Skewness	16.9 (3.99, 122.0)	.001		
	T2 DISCRETIZED HISTO ExcessKurtosis	0.12 (0.02, 0.64)	.022		
	ADC GLRLM SRE	0.10 (0.02, 0.30)	<.001		

OR, odds ratio; CI, confidence interval; AIC, Akaike Information Criterion; Accuracy from 10-fold cross-validation with 5 repeats

Table 4. N Stage (N1) Final Logistic Regression Model Estimates and Accuracy from Training Data.

Model	Variables	OR (95% CI)	*P* value	AIC	Accuracy, Mean (Range)
Clinical	Age, years	1.03 (0.98, 1.10)	.25	140	0.620 (0.400, 0.900)
	PSA, ng/ml	1.02 (1.01, 1.04)	.023		
T2w	T2 CONVENTIONAL Skewness	1.13 (0.54, 2.39)	.75	123	0.668 (0.364, 0.909)
	T2 CONVENTIONAL Kurtosis	1.90 (0.64, 6.29)	.27		
	T2 GLRLM LRE	3.75 (1.38, 12.2)	.017		
PET	PET GLRLM LRHGE	1.52 (0.69, 5.24)	.41	115	0.727 (0.444, 1.000)
	PET70 DISCRETIZED TLG mL only for PET or NM	11.4 (2.34, 76.1)	.006		
Clinical + T2w	Age, years	1.00 (0.94, 1.08)	.89	125	0.654 (0.300, 1.000)
	PSA, ng/ml	1.01 (1.00, 1.03)	.24		
	T2 CONVENTIONAL Skewness	1.12 (0.52, 2.45)	.78		
	T2 CONVENTIONAL Kurtosis	1.80 (0.61, 5.91)	.31		
	T2 GLRLM LRE	3.38 (1.19, 11.1)	.031		
Clinical + PET	Age, years	1.03 (0.97, 1.11)	.31	117	0.721 (0.444, 1.000)
	PSA, ng/ml	1.01 (1.00, 1.02)	.32		
	PET GLRLM LRHGE	1.57 (0.73, 5.42)	.36		
	PET70 DISCRETIZED TLG mL only for PET or NM	7.76 (1.62, 54.3)	.021		
T2w + PET	T2 CONVENTIONAL Skewness	0.90 (0.37, 2.19)	.82	99.0	0.773 (0.500, 1.000)
	T2 CONVENTIONAL Kurtosis	4.01 (1.06, 19.4)	.06		
	T2 GLRLM LRE	2.17 (0.67, 8.97)	.26		
	PET GLRLM LRHGE	2.08 (1.00, 7.08)	.13		
	PET70 DISCRETIZED TLG mL only for PET or NM	4.89 (1.02, 30.9)	.07		
Full	Age, years	0.95 (0.87, 1.04)	.27	102	0.739 (0.500, 1.000)
	PSA, ng/ml	1.00 (0.98, 1.02)	.86		
	T2 CONVENTIONAL Skewness	1.02 (0.41, 2.60)	.96		
	T2 CONVENTIONAL Kurtosis	4.74 (1.17, 25.5)	.045		
	T2 GLRLM LRE	2.09 (0.56, 9.08)	.31		
	PET GLRLM LRHGE	2.04 (1.02, 6.59)	.11		
	PET70 DISCRETIZED TLG mL only for PET or NM	5.53 (1.04, 39.3)	.06		

OR, odds ratio; CI, confidence interval; AIC, Akaike Information Criterion; Accuracy from 10-fold cross-validation with 5 repeats

Table 5. Sensitivity, Specificity, Positive and Negative Predictive Values, and AUC of Models Evaluated on Test Data.

Outcome	Model	Sensitivity (95% CI)	Specificity (95% CI)	PPV (95% CI)	NPV (95% CI)	AUC (95% CI)
ISUP GG ≥ 2	Clinical	0.654 (0.443, 0.828)	0.500 (0.068, 0.932)	0.895 (0.669, 0.987)	0.182 (0.023, 0.518)	0.673 (0.483, 0.863)
	T2w	0.769 (0.564, 0.910)	1.000 (0.398, 1.000)	1.000 (0.832, 1.000)	0.400 (0.122, 0.738)	0.914 (0.801, 1.000)
	ADC	0.500 (0.299, 0.701)	0.500 (0.068, 0.932)	0.867 (0.595, 0.983)	0.133 (0.017, 0.405)	0.567 (0.332, 0.803)
	PET	0.885 (0.698, 0.976)	0.250 (0.006, 0.806)	0.885 (0.698, 0.976)	0.250 (0.006, 0.806)	0.664 (0.215, 1.000)
	Clinical + T2w	0.846 (0.651, 0.956)	0.750 (0.194, 0.994)	0.957 (0.781, 0.999)	0.429 (0.099, 0.816)	0.885 (0.695, 1.000)
	Clinical + ADC	0.654 (0.443, 0.828)	0.250 (0.006, 0.806)	0.850 (0.621, 0.968)	0.100 (0.003, 0.445)	0.596 (0.387, 0.806)
	Clinical + PET	0.846 (0.651, 0.956)	0.500 (0.068, 0.932)	0.917 (0.730, 0.990)	0.333 (0.043, 0.777)	0.664 (0.285, 1.000)
	T2w + ADC	0.731 (0.522, 0.884)	0.750 (0.194, 0.994)	0.950 (0.751, 0.999)	0.300 (0.067, 0.652)	0.827 (0.651, 1.000)
	T2w + PET	0.962 (0.804, 0.999)	0.500 (0.068, 0.932)	0.926 (0.757, 0.991)	0.667 (0.094, 0.992)	0.981 (0.937, 1.000)
	ADC + PET	0.885 (0.698, 0.976)	0.250 (0.006, 0.806)	0.885 (0.698, 0.976)	0.250 (0.006, 0.806)	0.577 (0.180, 0.974)
	T2w + ADC + PET	0.885 (0.698, 0.976)	0.500 (0.068, 0.932)	0.920 (0.740, 0.990)	0.400 (0.053, 0.853)	0.856 (0.659, 1.000)
	Clinical + T2w + ADC	0.808 (0.606, 0.934)	0.500 (0.068, 0.932)	0.913 (0.720, 0.989)	0.286 (0.037, 0.710)	0.846 (0.671, 1.000)
	Clinical + T2w + PET	0.923 (0.749, 0.991)	0.750 (0.194, 0.994)	0.960 (0.796, 0.999)	0.600 (0.147, 0.947)	0.923 (0.766, 1.000)
	Clinical + ADC + PET	0.846 (0.651, 0.956)	0.250 (0.006, 0.806)	0.880 (0.688, 0.975)	0.200 (0.005, 0.716)	0.615 (0.265, 0.966)
	Full	0.923 (0.749, 0.991)	0.500 (0.068, 0.932)	0.923 (0.749, 0.991)	0.500 (0.068, 0.932)	0.885 (0.730, 1.000)
M Stage (M1)	Clinical	0.000 (0.000, 0.842)	0.750 (0.551, 0.893)	0.000 (0.000, 0.410)	0.913 (0.720, 0.989)	0.500 (0.000, 1.000)
	T2w	0.500 (0.013, 0.987)	0.750 (0.551, 0.893)	0.125 (0.003, 0.527)	0.955 (0.772, 0.999)	0.786 (0.356, 1.000)
	ADC	1.000 (0.158, 1.000)	0.714 (0.513, 0.868)	0.200 (0.025, 0.556)	1.000 (0.832, 1.000)	0.875 (0.675, 1.000)
	Clinical + T2w	0.500 (0.013, 0.987)	0.821 (0.631, 0.939)	0.167 (0.004, 0.641)	0.958 (0.789, 0.999)	0.768 (0.368, 1.000)
	Clinical + ADC	0.500 (0.013, 0.987)	0.714 (0.513, 0.868)	0.111 (0.003, 0.482)	0.952 (0.762, 0.999)	0.393 (0.000, 1.000)
	T2w + ADC	0.500 (0.013, 0.987)	0.929 (0.765, 0.991)	0.333 (0.008, 0.906)	0.963 (0.810, 0.999)	0.929 (0.774, 1.000)
	Full	0.000 (0.000, 0.842)	0.893 (0.718, 0.977)	0.000 (0.000, 0.708)	0.926 (0.757, 0.991)	0.821 (0.669, 0.974)

CI, confidence interval; PPV, positive predictive value; NPV, negative predictive value; AUC, area under the receiver operating characteristic curve

Click here to access/download;Figure;Figure 1_NEW.tiff

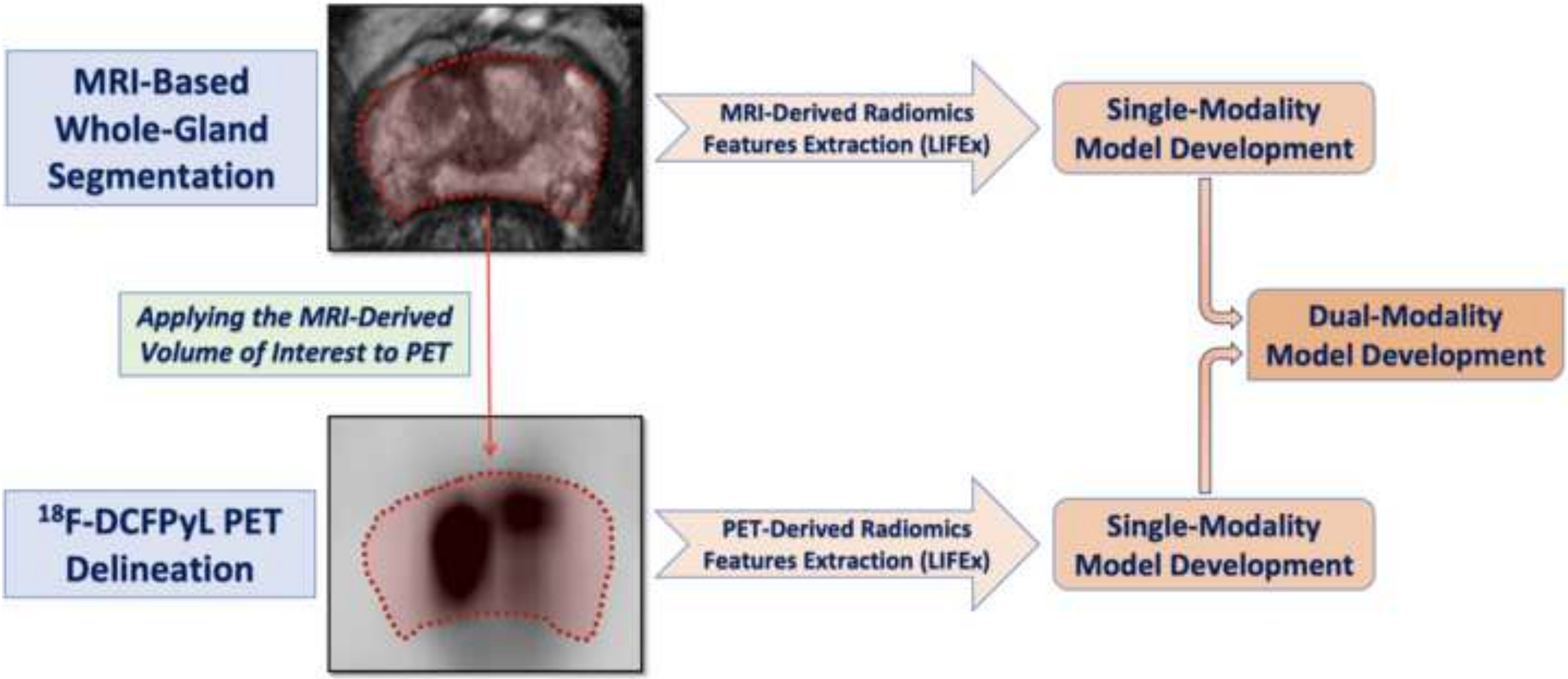

Figure XX-1

Click here to access/download
Supplementary material
XX-1.tiff

Figure XX-2

Click here to access/download
Supplementary material
XX-2.tiff

Click here to access/download
Supplementary material
Supp_CLEAN.docx
